# Catabolism of the Last Two Steroid Rings in *Mycobacterium tuberculosis* and Other Bacteria

**DOI:** 10.1128/mBio.00321-17

**Published:** 2017-04-04

**Authors:** Adam M. Crowe, Israël Casabon, Kirstin L. Brown, Jie Liu, Jennifer Lian, Jason C. Rogalski, Timothy E. Hurst, Victor Snieckus, Leonard J. Foster, Lindsay D. Eltis

**Affiliations:** a Department of Microbiology and Immunology, The University of British Columbia, Vancouver, Canada; b Department of Biochemistry and Molecular Biology, Life Sciences Institute, The University of British Columbia, Vancouver, Canada; c Centre for High-Throughput Biology (CHiBi), Department of Biochemistry and Molecular Biology, The University of British Columbia, Vancouver, Canada; d Department of Chemistry, Queen’s University, Kingston, Canada; Harvard School of Public Health

**Keywords:** CoA thioester, *Mycobacterium tuberculosis*, catabolism, cholesterol, ring opening

## Abstract

Most mycolic acid-containing actinobacteria and some proteobacteria use steroids as growth substrates, but the catabolism of the last two steroid rings has yet to be elucidated. In *Mycobacterium tuberculosis*, this pathway includes virulence determinants and has been proposed to be encoded by the KstR2-regulated genes, which include a predicted coenzyme A (CoA) transferase gene (*ipdAB*) and an acyl-CoA reductase gene (*ipdC*). In the presence of cholesterol, Δ*ipdC* and Δ*ipdAB* mutants of either *M. tuberculosis* or *Rhodococcus jostii* strain RHA1 accumulated previously undescribed metabolites: 3aα-*H*-4α(carboxyl-CoA)-5-hydroxy-7aβ-methylhexahydro-1-indanone (5-OH HIC-CoA) and (*R*)-2-(2-carboxyethyl)-3-methyl-6-oxocyclohex-1-ene-1-carboxyl-CoA (COCHEA-CoA), respectively. A Δ*fadE32* mutant of *Mycobacterium smegmatis* accumulated 4-methyl-5-oxo-octanedioic acid (MOODA). Incubation of synthetic 5-OH HIC-CoA with purified IpdF, IpdC, and enoyl-CoA hydratase 20 (EchA20), a crotonase superfamily member, yielded COCHEA-CoA and, upon further incubation with IpdAB and a CoA thiolase, yielded MOODA-CoA. Based on these studies, we propose a pathway for the final steps of steroid catabolism in which the 5-member ring is hydrolyzed by EchA20, followed by hydrolysis of the 6-member ring by IpdAB. Metabolites accumulated by Δ*ipdF* and Δ*echA20* mutants support the model. The conservation of these genes in known steroid-degrading bacteria suggests that the pathway is shared. This pathway further predicts that cholesterol catabolism yields four propionyl-CoAs, four acetyl-CoAs, one pyruvate, and one succinyl-CoA. Finally, a Δ*ipdAB M. tuberculosis* mutant did not survive in macrophages and displayed severely depleted CoASH levels that correlated with a cholesterol-dependent toxicity. Our results together with the developed tools provide a basis for further elucidating bacterial steroid catabolism and virulence determinants in *M. tuberculosis.*

## IMPORTANCE

Bacteria are the only known steroid degraders, but the pathway responsible for degrading the last two steroid rings has yet to be elucidated. In *Mycobacterium tuberculosis*, this pathway includes virulence determinants. Using a series of mutants in *M. tuberculosis* and related bacteria, we identified a number of novel CoA thioesters as pathway intermediates. Analysis of the metabolites combined with enzymological studies establishes how the last two steroid rings are hydrolytically opened by enzymes encoded by the KstR2 regulon. Our results provide experimental evidence for novel ring-degrading enzymes, significantly advance our understanding of bacterial steroid catabolism, and identify a previously uncharacterized cholesterol-dependent toxicity that may facilitate the development of novel tuberculosis therapeutics.

## INTRODUCTION

Steroids have a variety of important physiological roles across all domains of life. In eukaryotes, they serve as critical components of cell membranes, as signaling molecules, and in mammals, they absorb dietary fat. However, the only known organisms that can catabolize steroids and utilize them as growth substrates are bacteria (1). Bacterial steroid catabolism can be either aerobic, as occurs in most mycolic acid-containing actinobacteria and some proteobacteria (1–3), or anaerobic, as in some proteobacteria (4). Aerobic catabolism has been studied for many decades, due in part to its potential to transform low-value steroids into high-value ones (5). Most recently, the cholesterol catabolic pathway of *Mycobacterium tuberculosis* has been studied due to its role in virulence (2, 6, 7): this catabolism is required for the survival of *M. tuberculosis* in macrophages and is a potential target for novel therapeutics that are urgently needed to treat tuberculosis (8). Despite the intensified research, many aspects of steroid catabolism remain unclear.

**FIG 1:**
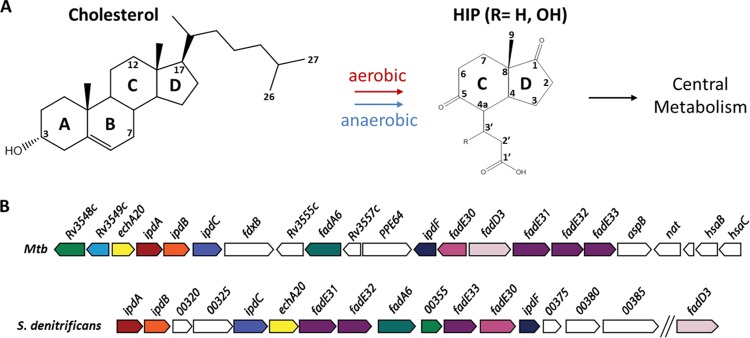
HIP catabolic genes in representative actinobacteria and proteobacteria. (A) The aerobic catabolism and anaerobic catabolism of cholesterol and other steroids appear to converge at HIP-CoA. (B) The HIP catabolic gene clusters of *M. tuberculosis* (*Mtb*) H37Rv and *S. denitrificans* DSMZ18526. Homologous genes are colored the same. For gene annotation, see Table 1.

Based on studies in *Rhodococcus jostii* RHA1, *M. tuberculosis*, and *Comamonas testosteroni* TA441, the aerobic catabolism of steroids largely follows the structural elements of the steroid molecule: the alkyl side chain when present, rings A and B, and rings C and D, respectively (3, 9, 10). Side-chain degradation resembles the β-oxidation of fatty acids (11) proceeding via coenzyme A (CoA) thioester intermediates to generate propionyl- and acetyl-CoA. Ring A/B degradation includes oxygenases that catalyze the 9,10 cleavage of the steroid nucleus and the 4,5-extradiol cleavage of ring A, respectively (12–14). In *M. tuberculosis* and other actinobacteria, genes encoding cholesterol uptake and side-chain and ring A/B degradation are transcriptionally regulated by KstR, a TetR family repressor (15), and side-chain and ring A/B degradation occur concurrently to at least some extent (16). In all aerobic steroid-degrading bacteria characterized to date, catabolism yields 3aα-*H*-4α(3′-propanoate)-7aβ-methylhexahydro-1,5-indanedione (HIP) (17, 18), a 13-carbon catabolite containing intact rings C and D or a derivative thereof (Fig. 1A).

In mycobacteria and rhodococci, HIP catabolism is specified by a series of enzymes encoded by ~15 genes that are transcriptionally regulated by a second TetR family repressor, KstR2 (10, 15) (Fig. 1B). However, in contrast to the catabolism of the steroid side chain and rings A and B, the catabolism of HIP remains largely uncharacterized. Fifty years ago, Lee and Sih proposed that HIP catabolism proceeds via the cleavage of ring C, involving either a β-oxidative reaction or a Baeyer-Villiger type mono-oxygenation (18). Subsequently, Hashimoto and Hayakawa (1977) reported the accumulation of 4-methyl-5-oxooctanedioic acid (MOODA) during the growth of a streptomycete on HIP (19). Homologs of the enzymes encoded by the KstR2 regulon have been found in all steroid-degrading bacteria characterized to date (Table 1). Actinobacteria that contain multiple steroid catabolic pathways appear to complete this catabolism using a single HIP catabolic pathway (9). Interestingly, recent bioinformatic and transcriptomic studies indicate that the aerobic and anaerobic steroid degradation pathways of *Steroidobacter denitrificans* also converge at HIP (4) (Fig. 1B). These studies in diverse bacteria suggest that a single HIP catabolic pathway is used in the bacterial catabolism of steroids. Nevertheless, the only characterized HIP catabolic enzyme to date is FadD3, which initiates catabolism by catalyzing the thioesterification of HIP in *M. tuberculosis* (17) and *Pseudomonas putida* DOC21 (20). In *Actinobacteria*, the reaction product HIP-CoA is the effector of KstR2 (10). Bioinformatic analyses suggest the involvement of at least two rounds of β-oxidation in the subsequent catabolism of HIP (17).

**TABLE 1:** Annotation of the KstR2 regulon

Gene^a^	Identification no. for^b^:					Annotation of gene product	Best hit^c^	% amino acid identity^d^
	H37Rv	RHA1	*M. smegmatis*	CNB-2	*S. denitrificans*			
	Rv3548c	RS22710	5999	1286	00355	Short-chain-type dehydrogenase/reductase	P22414	42
	Rv3549c	RS22705	6000	1330	12450^e^	Short-chain-type dehydrogenase/reductase	A6CQL2	34
*echA20*	Rv3550	RS27700	6001	1280	00335	HIEC-CoA hydrolase	P76082 (1,4-dihydroxy-2-naphthoyl-CoA synthase [MenB])	28
*ipdA*	Rv3551	RS22695	6002	1276	00310	COCHEA-CoA hydrolase, α subunit	Q59111 (glutaconate CoA-transferase, α subunit)	26
*ipdB*	Rv3552	RS22690	6003	1277	00315	COCHEA-CoA hydrolase, β subunit	Q59111 (glutaconate CoA-transferase, β subunit)	25
*ipdC*	Rv3553	RS22685	6004	1279	00330	5-OH HIC-CoA reductase	Q9FBC5 [enoyl-(ACP) reductase II (FabK)]	30
*fadA6*	Rv3556c	RS22430	6008	1283	00350	β-Keto CoA thiolase	I6XHI4 (3-oxo-acyl CoA thiolase)	38
*kstR2*	Rv3557c	RS22425	6009			HIP-CoA repressor^f^		
*ipdF*	Rv3559c	RS22420	6011	1289	00370	5-Oxo HIC-CoA oxidase	Q9LBG2 (levodione reductase)	39
*fadE30*	Rv3560c	RS22415	6012	1288	00365	Acyl-CoA dehydrogenase	I6YCA3	31
*fadD3*	Rv3561	RS22410	6013	1360	09100^e^	HIP-CoA synthetase^f^		
*fadE31*	Rv3562	RS22400	6014	1281	00340	Acyl-CoA dehydrogenase	I6YCA3	29
*fadE32*	Rv3563	RS22395	6015	1282	00345	MOODA-CoA dehydrogenase	I6YCA3	23
*fadE33*	Rv3564	RS22390	6016	1287	00360	Acyl-CoA dehydrogenase	I6YCA3	23

a Name assigned based on the present study.

b Identification numbers for the corresponding genes in *M. tuberculosis* H37Rv, *R. jostii* RHA1, *M. smegmatis*, *C. testosteroni* CNB-2, and *Steroidobacter denitrificans* DSM 18526. For simplicity, “Msmeg_” was omitted from identification numbers for *M. smegmatis*, “CtCNB2_” was omitted from those for *C. testosteroni* CNB-2, and “ACG33_” was omitted from those for *S.  denitrificans* DSM 18526.

c Accession numbers of functionally characterized best hits in the NCBI database are shown, with alternate protein names included for some entries.

d Amino acid sequence identity of the *M. tuberculosis* enzyme and its experimentally characterized best hit based on full sequence alignment.

e Not clustered with other ring C and D catabolic genes.

f *M. tuberculosis* enzyme characterized.

Transposon mutagenesis studies of the cholesterol catabolic genes in *M. tuberculosis* indicate that some of the intermediates of HIP catabolism are toxic to the bacterial cell (21). In these studies, *fadD3* insertion mutants were recovered at a reasonably high frequency in cholesterol- versus glycerol-containing media, consistent with the ability of a *fadD3* deletion mutant to grow on the first half of the cholesterol molecule (17). In contrast, mutants of each of eight other KstR2 regulon genes, predicted to encode enzymes that act downstream of FadD3, including *ipdA* (*Rv3551*) and *ipdC* (*Rv3553*), were recovered at much lower frequencies. This result is consistent with the lack of the encoded enzymes inducing some form of toxicity in the presence of cholesterol. Genes of the KstR2 regulon predicted to be essential for virulence in transposon mutagenesis studies in macrophages and mice include *ipdA*, *ipdB*, *fadA6*, *fadE30*, and *fadE32* (22, 23). The *ipdA* gene is most consistently implicated in these studies, and *ipdAB* mutants have been patented as a live vaccine based on studies in *Rhodococcus equi* (24). *R. equi* appears to be fairly unique in having a second copy of some KstR2 regulon genes, including *ipdAB* and *fadA6* (24).

Herein, we elucidate key steps of HIP catabolism, including the order of opening of rings C and D. We generated a series of mutants in the KstR2-regulated genes of three mycolic acid-containing actinobacteria: *M. tuberculosis*, *Mycobacterium smegmatis*, and *R. jostii* RHA1. We used liquid chromatography-coupled mass spectrometry (LC-MS) to interrogate these mutants for the accumulation of intracellular CoA metabolites under different growth conditions. This led to the resolution of previously undescribed CoA thioesters that represent various HIP metabolites, including ring C/D-opened intermediates. The identity of key compounds was determined using ^1^H- and ^13^C-nuclear magnetic resonance (NMR) and chemical synthesis. We used purified enzymes from actinobacteria and a proteobacterium to generate CoA thioesters that were observed in the mutants. Finally, we investigated the phenotype of a Δ*ipdAB* mutant of *M. tuberculosis* in macrophages to understand its importance in intracellular survival. The results enable us to propose a bacterial HIP catabolism pathway.

## RESULTS

### The *ipdABC* genes are required for growth on cholesterol and HIP.

The KstR2 regulon has been strongly implicated in the catabolism of HIP in mycolic acid-containing actinobacteria (10). Because FadD3, encoded by the KstR2 regulon, initiates HIP catabolism, we hypothesized that the other regulon-encoded enzymes act downstream of FadD3. To further elucidate the steps of the HIP catabolic pathway, we initially focused on *ipdAB* and *ipdC* in *M. tuberculosis* (*Rv3551* to *Rv3553*) and *R. jostii* RHA1 (RHA1_*RS22695-22685*).

**FIG 2:**
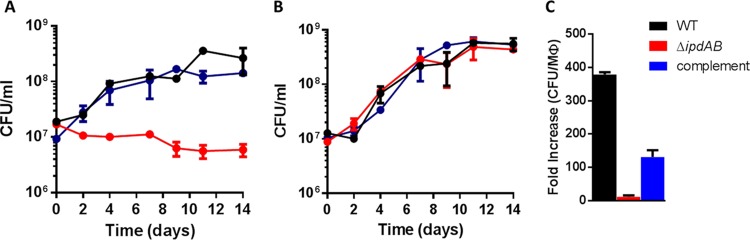
Growth of Δ*ipdAB M. tuberculosis*. WT *M. tuberculosis* Erdman (black), Δ*ipdAB M. tuberculosis* (red), or Δ*ipdAB*::*ipdAB M. tuberculosis* (blue) cells were grown on (A) 0.5 mM cholesterol, (B) on 0.2% glycerol, or (C) in phorbol myristate acetate (PMA)-differentiated THP-1 cells (MΦ). Data represent the mean from biological triplicates.

An Δ*ipdAB* mutant constructed in *M. tuberculosis* Erdman did not grow on cholesterol (Fig. 2A), but grew as the wild type (WT) on glycerol (Fig. 2B). The growth defect on cholesterol was restored through complementation. Because *M. tuberculosis* mutants can exhibit unexplained strain differences (25), we verified that an equivalent Δ*ipdAB* mutant constructed in *M. tuberculosis* CDC1551 had the same phenotype as the Δ*ipdAB M. tuberculosis* Erdman strain (data not shown). Finally, a Δ*ipdAB* mutant of *R. jostii* RHA1 exhibited a similar phenotype (see Fig. S1 in the supplemental material): it did not grow on either HIP or cholesterol and grew normally on pyruvate (Fig. S1). The growth defects on cholesterol and HIP were restored through complementation with *ipdAB* of *M. tuberculosis*.

10.1128/mBio.00321-17.2 FIG S1  Growth and CoA metabolites of *R. jostii* RHA1 strains. (A to D) Growth of WT::pTip-Qc2 (blue), Δ*ipdAB*::pTip-Qc2 (red), Δ*ipdAB*::pTipCoL51 (red, dashed), Δ*ipdC*::pTipQc2 (green), and Δ*ipdC*::pTipRv3553 (green, dashed) on (A) 10 mM pyruvate, (B) 1 mM cholesterol, (C) 1.5 mM HIP, and (D) 1 mM HIP plus 10 mM pyruvate. (E) Depletion of HIP by RHA1 strains, color coded as in growth curves as measured by GC-MS and reported as percentage of initial levels. Data are the mean from triplicates. Error bars show standard deviations. (F) LC-MS chromatograms of CoA metabolites extracted from WT (blue) and Δ*ipdAB* (red) RHA1 incubated with cholesterol. Numbers correspond to CoASH (1), acetyl-CoA (2), propionyl-CoA (3), and COCHEA-CoA (4). IS, internal standard. Data for panel D were acquired using a BioScreen C (Growth Curves USA). Download FIG S1, TIF file, 0.3 MB. Copyright © 2017 Crowe et al. 2017 Crowe et al. This content is distributed under the terms of the Creative Commons Attribution 4.0 International license.

**FIG 3:**
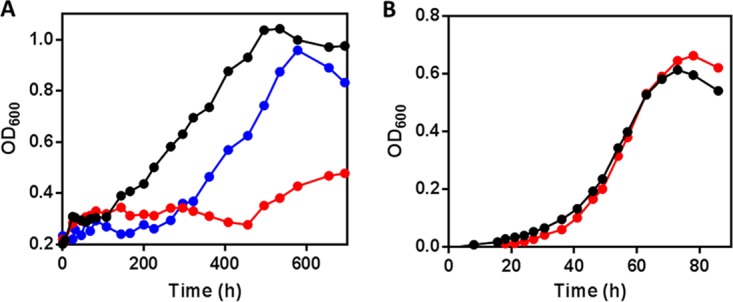
Growth of Δ*ipdC R. jostii* RHA1. WT RHA1::pTipQC2 (black), Δ*ipdC* RHA1::pTipQC2 (red), or Δ*ipdC* RHA1::pTipRv3553 (blue) cells were grown on (A) 1 mM cholesterol or (B) 10 mM sodium pyruvate. OD_600_, optical density at 600 nm.

An Δ*ipdC* mutant of *R. jostii* RHA1 also did not grow on either cholesterol or HIP but grew normally on pyruvate (Fig. 3; Fig. S1). The growth defect on cholesterol and HIP was restored through complementation with *M. tuberculosis ipdC* (*ipdC*_Mtb_). Gas chromatography-coupled mass spectrometry (GC-MS) established that while cholesterol and HIP were depleted in the wild-type and complemented strains, they were not detectably depleted by the *ipdC* mutant (Fig. S1E). Similar results were obtained in *M. tuberculosis* Erdman: the Δ*ipdC* mutant did not grow on cholesterol but grew normally on glycerol (see Fig. S2 in the supplemental material). Moreover, this phenotype was restored through complementation with an integrative plasmid harboring *ipdC*.

10.1128/mBio.00321-17.3 FIG S2  Growth and CoA metabolites of Δ*ipdC M. tuberculosis*. WT (black), Δ*ipdC* (red), and Δ*ipdC*::*ipdC* (blue) *M. tuberculosis* Erdman were grown on (A) 1 mM cholesterol, (B) 0.2% glycerol, or (C) 0.5 mM cholesterol and 0.2% glycerol. (D) CoA metabolome of Δ*ipdC* (red) and WT *M. tuberculosis* (blue) incubated with 0.5 mM cholesterol. Arrows indicate the peaks corresponding to the 5-OH HIC-CoA in the Δ*ipdC R. jostii* RHA1 CoA metabolome. Download FIG S2, TIF file, 0.3 MB. Copyright © 2017 Crowe et al. 2017 Crowe et al. This content is distributed under the terms of the Creative Commons Attribution 4.0 International license.

### Growth in macrophages.

Transposon mutagenesis studies suggest that *ipdA* is essential for *M. tuberculosis* survival in macrophages (23). Moreover, the gene is essential for survival of *R. equi* in foals (24). We therefore tested growth of Δ*ipdAB M. tuberculosis* in phorbol myristate acetate (PMA)-differentiated THP-1 cells (Fig. 2C). WT *M. tuberculosis* increased >350-fold over 7 days, corresponding to a doubling time of 19.6 h. The mutant increased ~10-fold over this time, corresponding to a doubling time of 46.5 h, while complementation restored intracellular replication to 131-fold. These results are consistent with *M. tuberculosis* catabolizing cholesterol during intracellular growth (8).

### Accumulation of cholesterol catabolites in the *ipd* mutants.

**FIG 4:**
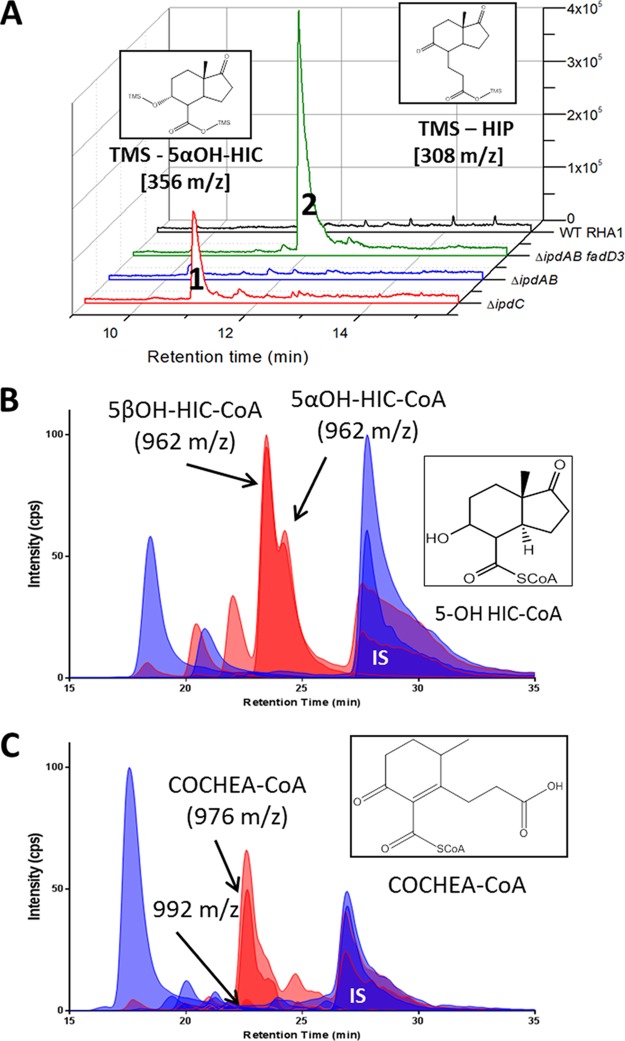
Accumulation of cholesterol-derived metabolites from Δ*ipdAB* and Δ*ipdC* strains. (A) GC-MS traces of culture supernatants of Δ*ipdC*, Δ*ipdAB*, Δ*fadD3 ipdAB*, and WT *R. jostii* RHA1 incubated with cholesterol. Peaks 1 and 2 correspond to TMS-5α-OH HIC and TMS-HIP, respectively. (B and C) CoA metabolome of cholesterol-incubated cells of (B) WT (blue) and Δ*ipdC* (red) RHA1 or (C) WT (blue) and Δ*ipdAB* (red) *M. tuberculosis*. The major unique peaks in the Δ*ipdC* and Δ*ipdAB* metabolomes correspond to 5αOH-HIC-CoA and COCHEA-CoA, respectively (inset). Lighter-shaded curves in panels B and C are based on the 768→261 transition observed in free CoASH as well as CoA thioesters subjected to in-source fragmentation.

In an attempt to identify the respective substrates of IpdAB and IpdC, we investigated the occurrence of metabolites in the RHA1 mutants. GC-MS analyses revealed that, when incubated with cholesterol, Δ*ipdC R. jostii* RHA1 accumulated small amounts of a metabolite with an *m*/*z* of 356 (Fig. 4A). No metabolites were detected in the culture supernatant when cells of Δ*ipdAB R. jostii* RHA1 were incubated with cholesterol (Fig. 4A).

We hypothesized that the failure to detect significant amounts of extracellular metabolites in the supernatants of cholesterol-incubated Δ*ipdC* and Δ*ipdAB* mutants was due to the accumulation of intracellular CoA-thioesterified metabolites that are not readily excreted. To test this hypothesis, we extracted the CoA thioesters from cells and analyzed them using liquid chromatography (LC)-MS. LC was performed using a pentafluorophenyl (PFP) resin to maximize resolution of the CoA thioesters. *M. tuberculosis*, *R. jostii* RHA1, and *M. smegmatis* cells incubated with various substrates contained CoASH, acetyl-CoA, propionyl-CoA, and/or succinyl-CoA irrespective of the growth substrate (see Table S2 in the supplemental material). The identity of these metabolites was based on their *m*/*z* values and their retention time (*R*_*t*_) on the PFP column with respect to synthetic standards. Their concentrations were quantified relative to *p*-coumaroyl-CoA, the internal standard. Some strains also contained small amounts of dephospho-CoASH, depending on the substrate, as has been reported in other cells (26).

10.1128/mBio.00321-17.8 TABLE S1  Strains, plasmids, and oligonucleotides used in this study. Download TABLE S1, DOCX file, 0.1 MB. Copyright © 2017 Crowe et al. 2017 Crowe et al. This content is distributed under the terms of the Creative Commons Attribution 4.0 International license.

10.1128/mBio.00321-17.9 TABLE S2  List and characterization of CoA thioesters in *M. tuberculosis*, *R. jostii* RHA1, *M. smegmatis*, and Δ*ipdAB* mutants. Download TABLE S2, DOCX file, 0.1 MB. Copyright © 2017 Crowe et al. 2017 Crowe et al. This content is distributed under the terms of the Creative Commons Attribution 4.0 International license.

When incubated in the presence of cholesterol, the *ipd* mutants accumulated CoA thioesters that were not detected in either the wild-type strains or the *ipd* mutants incubated with glycerol or pyruvate (see Fig. S3 in the supplemental material). More specifically, cholesterol-incubated cells of Δ*ipdC M. tuberculosis* and *R. jostii* RHA1 contained significant amounts of two CoA thioesters with *m*/*z* values of 962, one of which was more abundant than the other (Fig. 4B; Fig. S2D). The main CoA thioester that accumulated in cholesterol-incubated cells of Δ*ipdAB M. tuberculosis* and *R. jostii* RHA1 eluted with a *R*_*t*_ of 22.7 min and had an *m*/*z* value of 976 (Fig. 4C; Fig. S1F).

10.1128/mBio.00321-17.4 FIG S3  CoA thioesters and metabolites produced by *M. smegmatis* strains. (A) WT and (B) Δ*ipdAB* cells were incubated with cholesterol (blue) and glycerol (gray). Numbers represent CoASH (1), acetyl-CoA (2), succinyl-CoA (3), propionyl-CoA (4), unidentified CoA thioester of 838 *m*/*z* (5), unidentified CoA thioester of 852 *m*/*z* (6), and COCHEA-CoA (7). (C) GC-MS of culture supernatants of cholesterol-incubated *M. smegmatis* strains. Cells of the indicated strains were incubated with 0.5 mM cholesterol. Insets show structures of TMS-derivatized 5α-OH HIC and 2-(2-carboxyethyl)-3-methyl-6-oxocyclohex-1-ene-1. *, unidentified compounds present in all *M. smegmatis* extracts that are unrelated to cholesterol catabolism. Download FIG S3, TIF file, 0.4 MB. Copyright © 2017 Crowe et al. 2017 Crowe et al. This content is distributed under the terms of the Creative Commons Attribution 4.0 International license.

### Identification of CoA metabolites in the *ipd* mutants.

We predicted that the metabolite with an *m*/*z* value of 962 that accumulated in Δ*ipdC R. jostii* RHA1 and *M. tuberculosis* could be produced by the β-oxidative cleavage of acetyl-CoA from HIP-CoA and reduction of the 5-oxo group to yield 3aα-*H*-4α(3′-carboxyl-CoA)-5-hydroxy-7aβ-methylhexahydro-1-indanone (5-OH HIC-CoA) (Fig. 4B). To identify the metabolites that accumulated in the Δ*ipdC* mutants, the 5α and 5β isomers of 5-OH HIC were synthesized and confirmed by NMR (see the supplemental material). Each isomer was thioesterified to yield 5α- and 5β-OH HIC-CoA, respectively, which were then purified by high-performance liquid chromatography (HPLC). The high-resolution [M+H]^+^ *m*/*z* value and MS^3^ fragmentation pattern (of the [M+H]^+^ −507 fragment), of synthetic 5-OH HIC-CoA corresponded to those of the most abundant CoA thioesters in cholesterol-incubated Δ*ipdC* mutants. The presence of both α and β diastereomers was confirmed by the *R*_*t*_ values of their corresponding standards. Interestingly, the *m*/*z* value of the metabolite that accumulated in the supernatant of cholesterol-incubated Δ*ipdC R. jostii* RHA1 corresponds to that of 5-OH HIC (Fig. 4A).

The most abundant CoA thioester in cholesterol-incubated Δ*ipdAB* mutants accumulated in sufficient quantities to allow its isolation from the *R. jostii* RHA1 mutant for further characterization. The metabolite was identified as 2-(2-carboxyethyl)-3-methyl-6-oxocyclohex-1-ene-1-carboxyl-CoA (COCHEA-CoA) based on high-resolution mass spectrometry (976.1960 *m*/z), ^1^H-NMR, correlation spectroscopy (COSY)-NMR, total COSY (TOCSY)-NMR, heteronuclear multiple bond correlation (HMBC)-NMR, and heteronuclear single quantum coherence (HSQC)-NMR (see the supplemental material). Most diagnostically, the C-8 methyl protons appear as a doublet, establishing that C-6 bears a hydrogen and that ring D is open. C-1″ is thioesterified based on C-3′ having the same ^13^C-NMR chemical shift as in 2-(2-carboxyethyl)-3-methyl-6-oxocyclohex-1-ene-1 (27).

### Enzymatic transformation of 5-OH HIC-CoA.

To further elucidate the catabolism of HIP, we purified various KstR2 regulon-encoded enzymes, including IpdAB, IpdC, IpdF, EchA20, and FadA6. We used these preparations to evaluate their abilities to transform 5-OH HIC-CoA *in vitro*. We initially sought to work exclusively with the *M. tuberculosis* homologs. However, we were unable to obtain all of the *M. tuberculosis* homologs in stable, soluble forms despite testing various host strains and expression conditions. In some cases, the RHA1 homolog was more stable. For IpdC, we obtained the best preparations using the homolog from *P. putida* Doc21, a bile acid-degrading bacterium. The gene encoding *P. putida* IpdC (IpdC_Doc21_), DC0014-19, is the reciprocal best hit of *ipdC*_Mtb_ and occurs in a predicted operon similar in structure to that of *S. denitrificans*, which contains *ipdAB* (Fig. 1B). SDS-PAGE analyses of the various protein preparations are provided in Fig. S5 in the supplemental material. The subscripts “Mtb,” “RHA1,” and “Doc21” identify the parent species or strain of each enzyme. Transformation experiments were performed by incubating synthetic 5-OH HIC-CoA with various enzymes and characterizing the reaction products using LC-MS.

**FIG 5:**
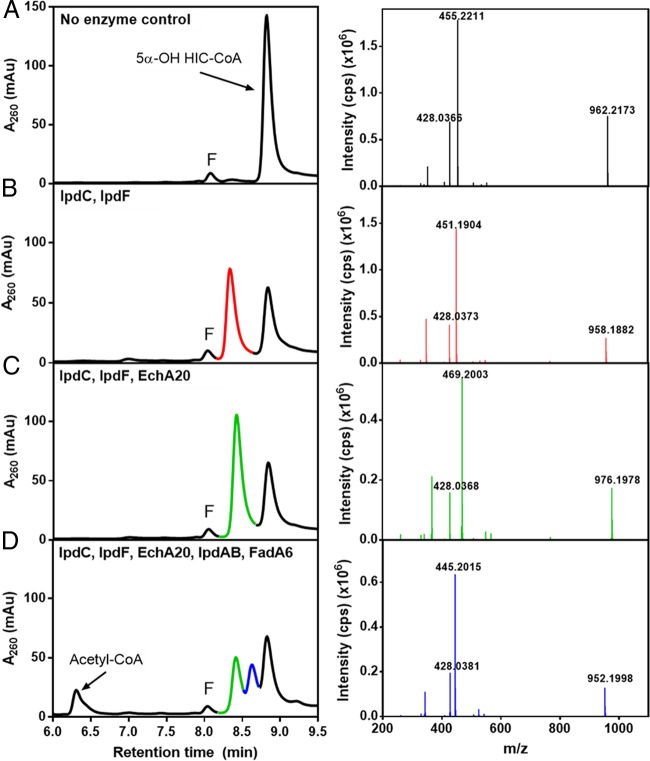
LC-MS analyses of the transformation of 5-OH HIC-CoA by purified enzymes. The left panels show HPLC traces of reaction mixtures containing 100 μM 5-OH HIC-CoA, 125 μM NAD^+^, 50 μM CoASH, 5 μM flavin mononucleotide (FMN) (10 mM phosphate [pH 7.5]) and (A) no enzyme (control), (B) IpdF_Mtb_ and IpdC_Doc21_, (C) IpdF_Mtb_, IpdC_Doc21_, and EchA20_RHA1_, or (D) IpdF_Mtb_, IpdC_Doc21_, EchA20_RHA1_, IpdAB_RHA1_, and FadA6_Mtb_. LC-MS analyses of the reaction products identified the major HPLC peaks. The major peaks are color coded with fragmentation patterns in the right-hand panels and correspond to (peak 1) 5α-OH HIC-CoA (962 *m*/*z*), (peak 2) HIEC-CoA (958 *m*/*z*), (peak 3) COCHEA-CoA (976 *m*/*z*), and (peak 4) MOODA-CoA (952 *m*/*z*). Other LC peaks correspond to acetyl-CoA (810 *m*/*z*) and FMN (labeled “F”).

Incubation of 5α-OH HIC-CoA with IpdF_Mtb_ and IpdC_Doc21_ yielded a compound with an *m*/*z* value of 958 (Fig. 5A and B; red trace), consistent with the oxidation of the 5-OH group of HIC and the introduction of a double bond. Interestingly, the two enzymes did not detectably transform 5β-OH HIC-CoA (data not shown). Based on its mass and the structure of the downstream metabolite, COCHEA-CoA, we provisionally identified the IpdF/IpdC transformation product as (7aS)-7a-methyl-1,5-dioxo-2,3,5,6,7,7a-hexahydro-1*H*-indene-4-carboxyl-CoA (HIEC-CoA). This assignment is consistent with the function of the next step of the pathway, as discussed below. However, NMR data are required for a definitive identification. Neither IpdF_Mtb_ nor IpdC_Doc21_ transformed 5-OH HIC-CoA in the absence of the other enzyme. Moreover, as described below, Δ*ipdF M. smegmatis* accumulated the same major metabolite as the Δ*ipdC* mutants, 5α-OH HIC-CoA. Therefore, we were unable to determine the order of reaction of IpdF and IpdC. However, incubation of 5-OH HIP-CoA with IpdF_Mtb_ yielded HIP-CoA, demonstrating that this enzyme catalyzes oxidation of the 5-OH.

Incubation of 5-OH HIC-CoA with IpdF_Mtb_, IpdC_Doc21_, and EchA20_RHA1_ yielded a compound whose *R*_*t*_ and *m/z* values were identical to those of COCHEA-CoA (Fig. 5C; green trace), the major metabolite that accumulated in the Δ*ipdAB* mutants.

**FIG 6:**
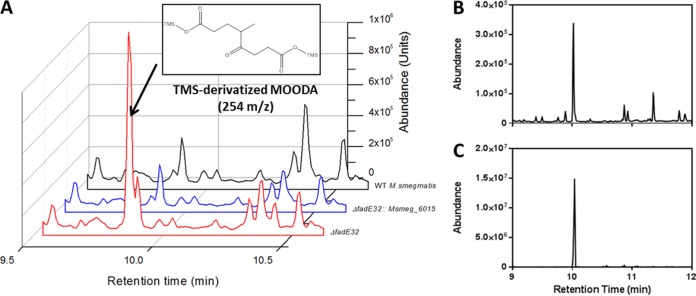
Cholesterol-derived metabolite of Δ*fadE32 M. smegmatis*. (A) GC-MS traces of culture supernatants of cholesterol-grown Δ*fadE32* (red), Δ*fadE32*::*msmeg_6015* (blue), and WT (black) *M. smegmatis*. The major metabolite observed in the mutant was MOODA (inset). (B) GC-MS trace of the product following hydrolysis of a metabolite of 952 *m*/*z* in 1 M NaOH. (C) MOODA purified from Δ*fadE32 M. smegmatis* incubated with cholesterol.

Finally, incubation of 5-OH HIC-CoA with IpdF_Mtb_, IpdC_Doc21_, EchA20_RHA1_, IpdAB_RHA1_, and FadA6_Mtb_ yielded a compound with an *m*/*z* value of 952 (Fig. 5D; blue trace). Hydrolysis of the CoA thioester yielded a compound that GC-MS revealed to be 4-methyl-5-oxo-octanedioc acid (MOODA), which accumulated in a Δ*fadE32* mutant of *M. smegmatis* when incubated with cholesterol (Fig. 6). MOODA-CoA has a predicted *m*/*z* value of 952. Consistent with such a role, the enzymatic transformation of COCHEA-CoA to MOODA-CoA required CoASH and yielded stoichiometric amounts of acetyl-CoA (Fig. 5D).

### Bioinformatic analysis of HIP catabolic enzymes.

To better understand the activities of IpdC, IpdF, EchA20, IpdAB, and FadA6, we performed bioinformatics analyses (Table 1). Among characterized homologs, IpdF shares 39% amino acid sequence identity with levodione reductase from *Corynebacterium aquaticum* M-13 (28), which catalyzes the NADH-dependent reduction of a ring ketone. For its part, IpdC shares 30% amino acid sequence identity with FabK from *Streptococcus pneumoniae*, an enoyl-acyl ACP reductase that catalyzes double-bond reduction (29), the reverse of the predicted IpdC reaction. Like FabK, purified IpdC_Doc21_ contained a flavin (data not shown). Overall, these analyses are consistent with the ability of IpdF and IpdC to catalyze the transformation of 5α-OH HIC-CoA to HIEC-CoA.

EchA20 is one of 21 EchAs in *M. tuberculosis*. EchAs are members of the crotonase superfamily that are predicted to catalyze the hydration of enoyl-CoAs (30), of which HIEC-CoA, the substrate of EchA20, is an example. A phylogenetic analysis revealed that among *M. tuberculosis* EchAs, only EchA20 clustered with MenB (see Fig. S4 in the supplemental material), although it shares ~28% amino acid sequence identity with each of the proteins EchA8, EchA18, and MenB. EchA8 and EchA18 are uncharacterized. However, MenB is a 1,4-dihydroxy-2-naphthoyl-CoA synthase that catalyzes an intramolecular Claisen condensation (Dieckmann cyclization) in menaquinone biosynthesis (31). This reaction is essentially the reverse of the reaction catalyzed by EchA20. EchA20 also shares 24% amino acid sequence identity with BadI of *Rhodopseudomonas palustris* (Table 1). BadI, a β-ketocyclohexanecarboxyl-CoA hydrolase involved in the anaerobic catabolism of benzoate (32), catalyzes a hydrolytic ring-opening reaction similar to that of EchA20. Overall, the bioinformatic analyses indicate that EchA20 catalyzes the hydrolytic ring opening of HIEC-CoA to COCHEA-CoA via a reverse Dieckmann cyclization. Nevertheless, it is unclear whether other *M. tuberculosis* EchAs catalyze similar reactions.

10.1128/mBio.00321-17.5 FIG S4  Phylogenetic tree of enoyl-CoA hydratases (EchAs) and MenB in *M. tuberculosis*. A cluster containing EchA20 and MenB_Mtb_ is highlighted with a red oval. Download FIG S4, TIF file, 0.1 MB. Copyright © 2017 Crowe et al. 2017 Crowe et al. This content is distributed under the terms of the Creative Commons Attribution 4.0 International license.

10.1128/mBio.00321-17.6 FIG S5  Electrophoretic analyses of gene deletion mutants and purified proteins. (A) PCR confirmation of Δ*ipdAB* in *R. jostii* RHA1 and *M. tuberculosis*, Δ*ipdC* in RHA1 and *M. tuberculosis*, and Δ*ipdF*, Δ*echA20*, and Δ*fadE32* in *M. smegmatis* using the listed primer sets (Table S1). (B) SDS-PAGE loaded, from left to right, with 0.5 μg each of MBP-IpdC_DOC21_, IpdF_Mtb_, EchA20_RHA1_, IpdAB_RHA1_, and FadA6_Mtb_. Purified proteins are flanked by molecular weight standards. Download FIG S5, TIF file, 0.4 MB. Copyright © 2017 Crowe et al. 2017 Crowe et al. This content is distributed under the terms of the Creative Commons Attribution 4.0 International license.

IpdAB shares 24% amino acid sequence identity with glutaconate CoA transferase (GCT) of *Acidaminococcus fermentans* (33), a type I CoA transferase. Most type I CoA transferases characterized to date catalyze the transfer of CoA between short acyl chains (33–36). To test whether IpdAB_RHA1_ had such activity, we incubated the enzyme with acetyl-CoA, propionyl-CoA, or succinyl-CoA in the presence of acetate, propionate, and succinate. In none of these instances was any CoA transferase activity detected. Finally, FadA6 shares 38% amino acid sequence identity with FadA5, a β-ketoacyl-CoA thiolase involved in cholesterol side-chain degradation (37) (Table 1), consistent with it catalyzing the thiolysis of a COCHEA-CoA ring-opened product containing a β-keto thioester moiety, to MOODA-CoA and acetyl-CoA.

### Validation of HIP catabolism using additional mutants.

**FIG 7:**
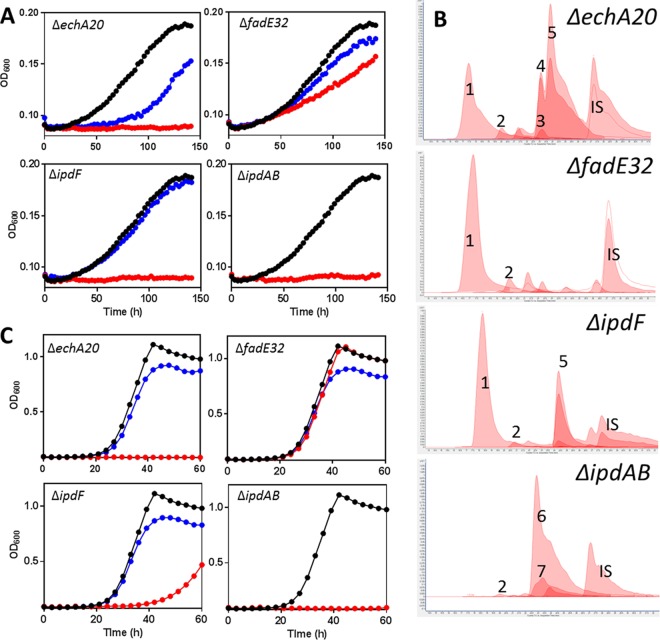
Characterization of KstR2 regulon mutants of *M. smegmatis*. Growth of Δ*echA20*, Δ*fadE32*, Δ*ipdF*, and Δ*ipdAB M. smegmatis* mutants on (A) 1.5 mM HIP or (C) 1 mM HIP plus 0.2% glycerol. Curves show WT (black), KstR2 regulon mutants (red), and corresponding complements (blue) and are the means from three biological replicates. (B) CoA metabolomes of mutants. The numbers correspond to CoASH (1), acetyl-CoA (2), HIEC-CoA (3), 5β-OH HIC-CoA (4), 5α-OH HIC-CoA (5), COCHEA-CoA (6), and unknown CoA thioester of 992 *m*/*z* (7). IS, *p*-coumaroyl-CoA internal standard. Lighter-shaded curves indicate the 768→261 transition (Fig. 4).

To obtain further evidence for the HIP catabolic pathway suggested by analyses of the *ipd* mutants and the enzymatic transformations of 5-OH HIC-CoA, we deleted the following KstR2-regulated genes in *M. smegmatis* MC^2^155 and analyzed the Δ*ipdF*, Δ*ipdAB*, Δ*echA20*, and Δ*fadE32* mutants (*msmeg_6011*, *msmeg_6002-6003*, *msmeg_6001*, and *msmeg_6015*, respectively). All four mutants were defective for growth on HIP: the Δ*fadE32* strain grew more slowly on HIP, while the other three did not grow at all (Fig. 7A). The growth defect of each mutant on HIP was complemented by the *M. tuberculosis* or *M. smegmatis* gene supplied in *trans*. GC-MS analysis of the culture supernatants revealed that the Δ*ipdF* and Δ*echA20* mutants accumulated small amounts of 5-OH HIC (Fig. S3C). Moreover, the Δ*fadE32* mutant accumulated a metabolite whose trimethylsilane (TMS) derivative had an *m*/*z* value of 346 (Fig. 6). The metabolite that accumulated in the supernatant of the cholesterol-grown Δ*fadE32* mutant was purified and was identified as MOODA based on ^1^H-, COSY-, HMBC-, and HSQC-NMR analysis (SI).

The Δ*ipdAB* mutant of *M. smegmatis* differed from those of *R. jostii* RHA1 and *M. tuberculosis* in that it accumulated two major metabolites in the supernatant when incubated with cholesterol. These had *m*/*z* values of 254 and 326 when derivatized with TMS reagent (Fig. S3C). The former was purified, and based on ^1^H-NMR, was identified as an analog of COCHEA lacking the C1 carboxyl (SI). A similar result was reported in an *ipdAB* deletion mutant of *Comamonas testosteroni* (27).

Cholesterol-incubated cells of the Δ*ipdF*, Δ*echA20*, and Δ*fadE32* mutants were analyzed for CoA thioesters. The profile of the Δ*ipdF* mutant was similar to that of the Δ*ipdC* mutant, containing a significant amount of 5α-OH HIC-CoA and a lesser amount of 5β-OH HIC-CoA (Fig. 7B). This is consistent with the enzymatic studies inasmuch as neither IpdC nor IpdF alone significantly transformed 5-OH HIC-CoA. The CoA metabolome of Δ*echA20 M. smegmatis* also contained significant quantities of the 5-OH HIC-CoA (Fig. 7B). However, it also contained a small amount of a metabolite whose retention time and *m*/*z* value (958) corresponded to that of the transformation product of 5-OH HIC-CoA by IpdC and IpdF (Fig. 7B). The CoA metabolome of Δ*ipdAB M. smegmatis* was very similar to those of the corresponding *R. jostii* RHA1 and *M. tuberculosis* mutants (Fig. 7B). Finally, the CoA metabolome of the Δ*fadE32* mutant was indistinguishable from that of WT *M. smegmatis*.

### HIP-dependent toxicity.

**FIG 8:**
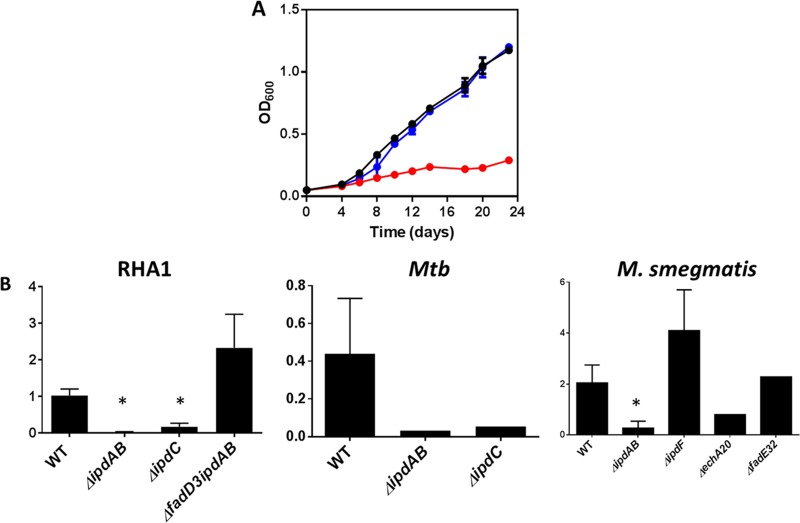
Cholesterol-dependent toxicity. (A) Growth of WT (black), Δ*ipdAB* (red), and Δ*ipdAB*::*ipdAB* (blue) *M. tuberculosis* grown on 7H9 medium containing 0.5 mM cholesterol and 0.2% glycerol. The data represent the average from biological triplicates. (B) The relative abundance of CoASH (768→261) was normalized to the internal standard (*p-*coumaroyl-CoA [914→407]) in KstR2 regulon mutants. *, *P* < 0.05 compared to the WT strain. Error bars represent standard deviations. The numbers of replicates were as follows: 5, 5, 5, and 3 for WT, Δ*ipdAB*, Δ*ipdC*, and Δ*fadD3* Δ*ipdAB R. jostii* RHA1, respectively; 2, 1, and 1 for WT, Δ*ipdAB*, and Δ*ipdC M. tuberculosis* (*Mtb*), respectively; and 4, 5, 1, 4, and 1 for WT, Δ*ipdAB*, Δ*echA20*, Δ*ipdF*, and Δ*fadE32 M. smegmatis*, respectively.

The failure of the Δ*ipdAB* and Δ*ipdC* mutants to grow on cholesterol (Fig. 2A, 3A, and 7A) is in marked contrast to the phenotype of Δ*fadD3* RHA1, which grows on cholesterol to ~50% the yield of the wild type (17). More specifically, the failure of the *ipd* mutants to grow on cholesterol despite the fact that the encoded enzymes act downstream of FadD3 suggests that the *ipd* deletions induce some form of toxicity. To explore this further, KstR2 regulon mutants were grown on a second carbon source in the presence of HIP. Interestingly, the Δ*fadE32* and Δ*ipdF* mutants grew on other carbon sources in the presence of HIP (Fig. 7C), while the Δ*ipdAB*, Δ*ipdC*, and Δ*echA20* mutants did not (Fig. 7C and 8A; Fig. S1D and S2C). The inability to catabolize a secondary carbon source in the presence of HIP indicates that there is a HIP-dependent toxicity in some of the mutants, similar to the cholesterol-dependent toxicity observed for the *ipdAB* and *ipdC* mutants described above. One possible form of cholesterol (or HIP)-dependent toxicity is the accumulation of propionyl-CoA, which can be relieved by supplementation with vitamin B_12_ (38). However, supplementation of the Δ*ipdAB*, Δ*ipdC*, and Δ*echA20* mutants with vitamin B_12_ did not relieve cholesterol-dependent toxicity, indicating that the basis of toxicity is independent of propionyl-CoA in these mutants. However, in analyzing the CoA metabolites of these mutants, we noted that the Δ*ipdAB*, Δ*ipdC*, and Δ*echA20* mutants contained significantly lower levels (<20%) of CoASH compared to the WT when cells were incubated with cholesterol (Fig. 8B). In contrast, the Δ*fadE32* and Δ*ipdF* mutants contained statistically similar CoASH levels to the WT under these conditions. Indeed, the respective levels of CoASH and cholesterol-derived CoA thioesters appeared to be inversely related. For example, when incubated with cholesterol, 5-OH HIC-CoA and COCHEA-CoA accounted for 84% ± 2% and 94% ± 1% of the total CoA detected in cells of Δ*ipdC* and Δ*ipdAB* RHA1, respectively, indicating that sequestration of CoASH by cholesterol-derived CoA-thioesters may be the basis of toxicity.

## DISCUSSION

**FIG 9:**
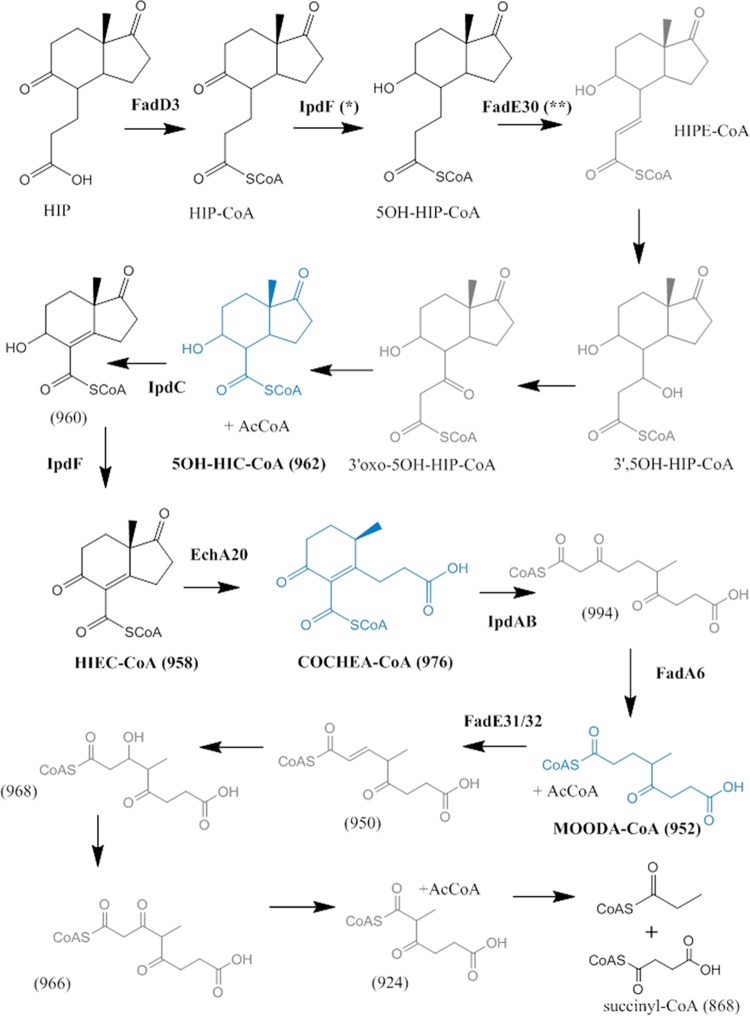
Proposed HIP catabolic pathway. NMR-confirmed metabolites are in blue. Metabolites for which MS data were obtained are in black. Other metabolites are in gray. *, the current study established that IpdF has this activity, but its physiological relevance is unclear. **, the role of FadE30 assigned previously (24).

The mutant data, enzymological transformations, and bioinformatic analyses presented herein support a model for HIP degradation in which cleavage of ring D precedes that of ring C (Fig. 9). More specifically, we propose a pathway for HIP catabolism in which the propionyl side chain is first degraded via β-oxidation to yield 5-OH HIC-CoA. This is then transformed to HIEC-CoA by IpdF and IpdC, before undergoing two successive ring cleavage reactions: EchA20-catalyzed hydrolysis of ring D followed by IpdAB-catalyzed hydrolysis of ring C. Thiolysis of the ring C-opened product, potentially by FadA6 or another thiolase, yields MOODA-CoA which is then oxidized to ^2^Δ-MOODA-CoA by an acyl-CoA dehydrogenase (ACAD) comprised in whole or in part by FadE32. Although the fate of ^2^Δ-MOODA-CoA is unclear, we propose that it undergoes a final round of β-oxidation to yield 2-methyl-β-ketoadipyl-CoA (MβKA-CoA). This could then be cleaved to propionyl-CoA and succinyl-CoA in a manner analogous to the cleavage of β-ketoadipyl-CoA to succinyl-CoA and acetyl-CoA in the final step of the β-ketoadipate pathway used in the bacterial catabolism of aromatic compounds (39). While several aspects of the HIP pathway have yet to be elucidated, three key metabolites have been definitively characterized: 5-OH HIC-CoA, COCHEA-CoA, and MOODA. Moreover, the data support the proposed physiological roles of four enzymes: IpdF, IpdC, EchA20, and IpdAB. The identity of the thiolase is less clear because FadA5 could be substituted for FadA6 (results not shown).

Among the enzymes whose functions were assigned, only that of IpdAB was unexpected with respect to the bioinformatic analyses (Table 1). More specifically, no type I CoA transferase has been reported to catalyze a retro-aldol hydrolysis. Nevertheless, two lines of evidence indicate that IpdAB is not a CoA transferase. First, IpdAB_RHA1_ did not catalyze the transfer of CoA between short-chain acyl substrates, in contrast to other type I CoA transferases characterized to date (33–36). Second, sequence alignments indicate that the catalytically essential glutamate in the β subunit of type I CoA transferases is not conserved in IpdAB, corresponding to Gly57^β^ in IpdAB_RHA1_. This glutamate, which is Glu54^β^ in GCT, forms an anhydride with CoA in the transferase reaction (33). Finally, although inclusion of IpdAB_RHA1_ in the reaction mixture containing IpdF_Mtb_, IpdC_Doc21_, and EchA20_RHA1_ did not detectably alter the reaction product, COCHEA-CoA, in the absence of IpdAB_RHA1_, the reaction only proceeded to ~10% completion. This suggests that interactions between the KstR2-encoded enzymes may accelerate the reactions.

The proposed catabolic pathway provides an important framework for further characterizing various aspects of steroid metabolism, including the identity of specific metabolites, such as HIEC-CoA and MβKA-CoA, as well as enzymatic steps, such as those catalyzed by Rv3548c and Rv3549c, encoded by the KstR2 regulon. The model further suggests the identities of the *fadE*-encoded ACADs that act on HIP-CoA and MOODA-CoA, respectively. Another unknown aspect of the pathway is the significance of the IpdF-catalyzed reaction: it is unclear why the 5-oxo group would be reduced and then reoxidized. Finally, this pathway also predicts that cholesterol feeds into central metabolism via four propionyl-CoAs, four acetyl-CoAs, one pyruvate, and one succinyl-CoA. Notably, propionyl-CoA, a potentially toxic metabolite (38), is derived from all three parts of cholesterol: the side chain, rings A and B, and rings C and D.

The HIP catabolic genes are conserved in steroid-degrading bacteria for which genome sequence data are available, suggesting that the pathway is employed not only in the degradation of steroids other than cholesterol (2, 3, 9), but also in the anaerobic degradation of steroids (4). Indeed, differences in the HIP catabolic gene cluster in diverse bacteria appear to reflect the different steroid-catabolizing capabilities of the strains. For example, in bacteria that catabolize cholate or other bile acids, the HIP catabolic gene cluster contains *echA13* (RHA1_RS22405 in *R. jostii* RHA1) (1, 9, 10). A homolog of EchA20, EchA13 is proposed to remove the hydroxyl of 7β-OH HIP, generated from cholate degradation (10, 20), and is not present in *M. tuberculosis*, which does not degrade cholate. Similarly, the HIP catabolic gene cluster of *S. denitrificans* DSM 18526, which is upregulated during the aerobic and anaerobic catabolism of testosterone (4), lacks a homolog of *fadD3*/*stdA3* (Fig. 1). Instead, this strain contains a homolog elsewhere in the genome: ACG33_09100 shares 53% amino acid sequence identity with StdA3 of *P. putida* DOC21 (20). The genomic context of *fadD3* in *S. denitrificans* DSM 18526 may reflect the possibility that the β-oxidation of steroid rings A and B yields HIP-CoA directly, obviating the need for FadD3 in anaerobic steroid catabolism.

Recent interest in bacterial steroid degradation has been fueled in large part by its role in the pathogenesis of *M. tuberculosis* (8, 40). Disruption of cholesterol catabolic genes generates both attenuated and avirulent strains of *M. tuberculosis* due to the predicted accumulation of toxic cholesterol-derived metabolites (7, 41, 42). Our findings corroborate this hypothesis. Deletion of *ipdAB* and *ipdC* in *M. tuberculosis* yielded strains that failed to grow on glycerol in the presence of cholesterol and, in the case of *ipdAB*, significantly slowed the growth in THP-1-derived macrophages. These strains displayed distinct differences in the concentration and identity of CoA thioesters and CoASH. This cholesterol-dependent toxicity of the mutants may be due to the sequestration of CoASH, making it unavailable for other cellular processes. Interestingly, disruption of the ratio between acetyl-CoA and propionyl-CoA in *M. tuberculosis* during growth on cholesterol has been reported to result in a toxic phenotype (43). Intriguingly, reduction in CoASH in strains displaying cholesterol-dependent toxicity typically coincided with reduced acetyl-CoA levels in our CoA metabolic data (data not shown). Consistent with the sequestration hypothesis, strains that grow in the presence of cholesterol (Δ*fadD3 R. jostii* RHA1, Δ*fadE32 M. smegmatis*, and Δ*ipdF M. smegmatis*) failed to accumulate significant levels of unique, cholesterol-derived CoA thioesters (17). The lack of toxicity in the Δ*ipdF* and Δ*fadE32* mutants could reflect the ease of hydrolysis of the corresponding CoA thioesters (e.g., MOODA-CoA to MOODA) and/or the presence of compensatory enzymatic activities (e.g., other cellular dehydrogenases could catalyze oxidation of the 5-OH HIC-CoA hydroxyl). Although additional data are required to test these hypotheses, the observed toxicity rationalizes the effectiveness of the *ipdAB* mutant as a vaccine in *R. equi* and indicates which HIP degradation enzymes would be good targets.

## MATERIALS AND METHODS

Additional materials and methods are provided in Text S1 in the supplemental material.

10.1128/mBio.00321-17.1 TEXT S1  Supplemental materials and methods. Download TEXT S1, DOCX file, 0.2 MB. Copyright © 2017 Crowe et al. 2017 Crowe et al. This content is distributed under the terms of the Creative Commons Attribution 4.0 International license.

### Preparation of CoA metabolites.

Cells were grown in 900 ml pyruvate or glycerol minimal medium as described above. Cells were harvested at mid-log phase, washed with fresh medium lacking growth substrate, and then suspended in 100 ml growth medium supplemented with either 0.5 mM cholesterol, 20 mM pyruvate (*R. jostii* RHA1), or 0.2% glycerol (*M. smegmatis* and *M. tuberculosis*). Biotransformants were incubated for 48 h at 30°C at 200 rpm for RHA1 and 37°C at 200 rpm for *M. smegmatis* and 37°C in roller bottles for *M. tuberculosis*. Cells were cooled on ice, harvested by centrifugation, washed once with ice-cold minimal medium, and then stored at −80°C until use.

CoA thioesters were extracted from cell pellets using a modified protocol described for eukaryotic cells (44). Preparations were kept on ice or 4°C unless otherwise noted. Frozen cell pellets were suspended in 4 ml of acetonitrile-isopropanol (3:1 vol/vol) containing 15 to 50 nmol *p*-coumaroyl-CoA. Cells were disrupted using a FastPrep-24 bead beater (6 × 40 s at 6.5 m/s, with 5-min pauses on ice between rounds). After the first three rounds, 0.1 M KH_2_PO_4_ (pH 6.7) was added to a final concentration of ~25 mM KH_2_PO_4_. The supernatant was recovered by centrifugation (15,000 × *g* for 30 min), filtered through a 0.2-µm-pore regenerated cellulose membrane (Phenomenex), and acidified with 0.25 ml glacial acetic acid per ml of extract. The acidified extract was applied to a 100 mg 2-(2-pyridyl)ethyl-functionalized silica column (Supelco, 54127-U), equilibrated using 1 ml “equilibration” solution (acetonitrile-isopropanol-water-acetic acid [9:3:4:4 vol/vol:vol/vol]) at −20°C. The resin was washed with 2 ml equilibration solution at −20°C before CoA metabolites were eluted with 2 ml methanol–0.25 M ammonium acetate (pH 7) (4:1 vol/vol) at −20°C. Methanol was evaporated under N_2_, and then the sample was flash frozen in liquid N_2_ and lyophilized overnight. The lyophilized sample was suspended in 0.6 ml methanol, deposited on a Phree column (Phenomenex) to remove phospholipids, and recovered by centrifugation (500 × *g* for 10 min). This sample eluate was dried under N_2_, suspended in 0.2 ml methanol, and stored at −80°C. Immediately prior to LC-MS analysis (described below), the cellular extracts was diluted 10-fold in 0.1 M ammonium acetate (pH 4.5) and filtered in a 0.2-µm-pore polytetrafluoroethylene (PTFE) filter.

### CoA metabolite profiling using MRM.

CoA thioesters were detected in cellular extracts using an Agilent 6460 triple quadrupole (QQQ) mass spectrometer operated in positive-ion mode and connected to an 80- by 0.25-mm Luna 3-μm PFP(2) (Phenomenex) analytical column through a 15- by 0.25-mm PFP(2) trapping column. CoA thioesters were separated using a gradient of 100 mM ammonium acetate (pH 4.5) into 20 mM ammonium acetate (pH 4.5) in 98% methanol over 30 min, operated at 3 μl min^−1^. A mixture of CoA thioester standards was run prior to each CoA metabolome to verify column performance and multiple reaction monitoring (MRM) sensitivity (see Fig. S6A in the supplemental material). Collision energy dissociation (CID) and fragmentor voltages were selected based on signal optimization using CoA thioester standards (Fig. S6). The MRM transitions recorded for each CoA metabolome are described below.

10.1128/mBio.00321-17.7 FIG S6  LC-MS of CoA thioester standards. (A) Peaks correspond to 25 pmol CoASH (light blue), acetyl-CoA (purple), propionyl-CoA (dark blue), 5α-OH HIC-CoA (yellow), HIP-CoA (green), and *p*-coumaroyl-CoA (red). (B) Representative standard curves for authentic CoA thioesters. Data points correspond to [M+H]^+^ → [M+H −507]^+^ (blue) and [M+H]^+^ → 428 (red) transitions. Lines represent best fit linear regression as follows: CoASH (768→261, *y* = 667.34*x* − 1,699.3, *R*^2^ = 0.9633; 768→428, *y* = 350.09*x* + 157.39, *R*^2^ = 0.9892), acetyl-CoA (810→303, *y* = 1,167.7*x* − 2,786.8, *R*^2^ = 0.9803; 810→428, *y* = 254.43*x* − 365.49, *R*^2^ = 0.9742), HIP-CoA (988→481, *y* = 447.29*x* − 1,099, *R*^2^ = 0.9734; 988→428, *y* = 97.542*x* − 192.62, *R*^2^ = 0.9735), *p-*coumaroyl-CoA (914→407, *y* = 428.8*x* + 19.13, *R*^2^ = 0.9786). (C) Collision energy dissociation (CID) optimization. Peak intensities of 50 pmol authentic CoA thioester standards for [M+H]^+^ → [M+H −507]^+^ (blue) and [M+H]^+^ → 428 (red) transitions over different CID voltages. Download FIG S6, TIF file, 0.4 MB. Copyright © 2017 Crowe et al. 2017 Crowe et al. This content is distributed under the terms of the Creative Commons Attribution 4.0 International license.

### MS/MS-based untargeted analysis.

To ensure that no CoA thioesters were missed using our targeted analysis method, representative CoA metabolomes were analyzed using LC-tandem MS (MS/MS) as previously described (26). Briefly, cellular extracts were diluted 1:49 with acetonitrile-water (3:97 vol/vol) supplemented with 0.1% formic acid and then injected onto a Zorbax SB300-C_18_ 150- by 0.075-mm column (Agilent Technologies) operated at 0.3 μl min^−1^ and eluted using a 10-min gradient from 3 to 97% acetonitrile. Mass spectra were recorded in positive-ion mode on an Agilent 6550 time of flight (ToF) mass spectrophotometer using a scanned mass range of 50 to 1,100 Da. Species were determined to be CoA thioesters based on the characteristic [M+H]^+^ −507 and 428 *m*/*z* fragments.

### High-resolution MS.

High-resolution MS, MS^2^, and MS^3^ analyses of CoA thioesters were performed in positive-ion mode on a Bruker Impact-II Q-ToF mass spectrometer equipped with a 150- by 0.25-mm Luna 3-μm PFP(2) (Phenomenex) column. CoA thioesters were eluted using a gradient of 100 mM ammonium acetate in 2% methanol and 20 mM ammonium acetate in 98% methanol. The mass spectrometer was calibrated daily.

### Analysis of CoA metabolomic data.

Peak integration, retention time, and the signal-to-noise (S/N) ratio were calculated using MassHunter Qualitative Analysis B.06.00 (Agilent Technologies). Peaks were defined as having an S/N ratio of >3. Analysis of CoA metabolomic data was completed using the [M+H]^+^ → [M+H −507]^+^ *m*/*z* transitions due to the higher signal intensity compared to the [M+H]^+^ → 428 *m*/*z* transitions, although the latter transition was confirmed for each CoA thioester characterized. CoA thioester levels were normalized to the internal standard (*p*-coumaroyl-CoA) prior to calculating their relative concentrations and proportion of the total cellular CoA pool. A complete summary of [M+H]^+^ → [M+H −507]^+^ transitions for each mutant is provided in Table S3 in the supplemental material.

10.1128/mBio.00321-17.10 TABLE S3  List of targeted MRMs followed for each CoA metabolome analyzed by LC-MS. Download TABLE S3, DOCX file, 0.1 MB. Copyright © 2017 Crowe et al. 2017 Crowe et al. This content is distributed under the terms of the Creative Commons Attribution 4.0 International license.
