# Supplementary figures and images for: Catabolism of the Last Two Steroid Rings in Mycobacterium tuberculosis and Other Bacteria
Source: mBio. 2017 Apr 4;8(2):e00321-17. doi: 10.1128/mBio.00321-17 (PMC5380842; doi:10.1128/mBio.00321-17)

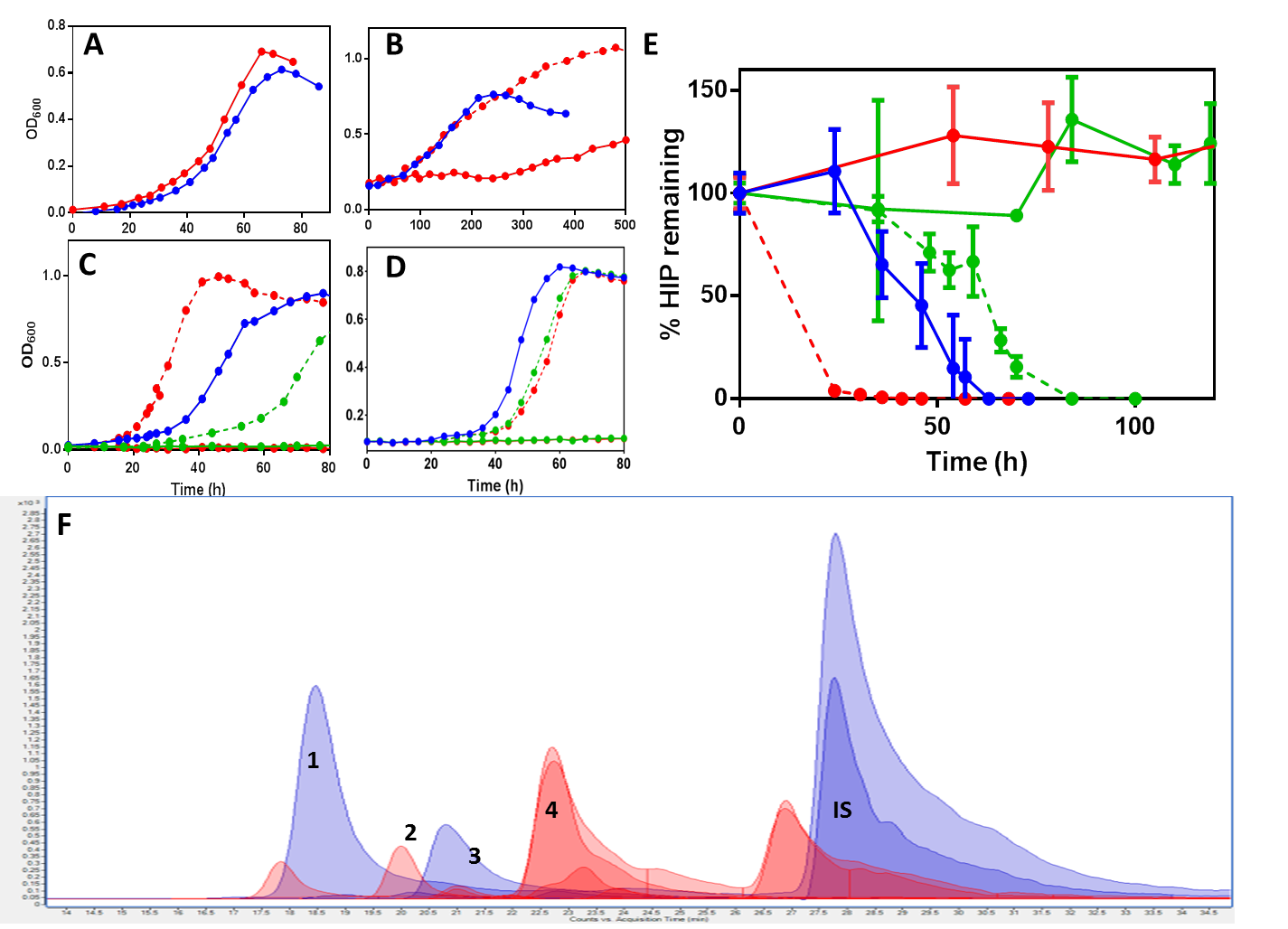

Supplement: FIG S1 [file mbo002173251sf1.tif]

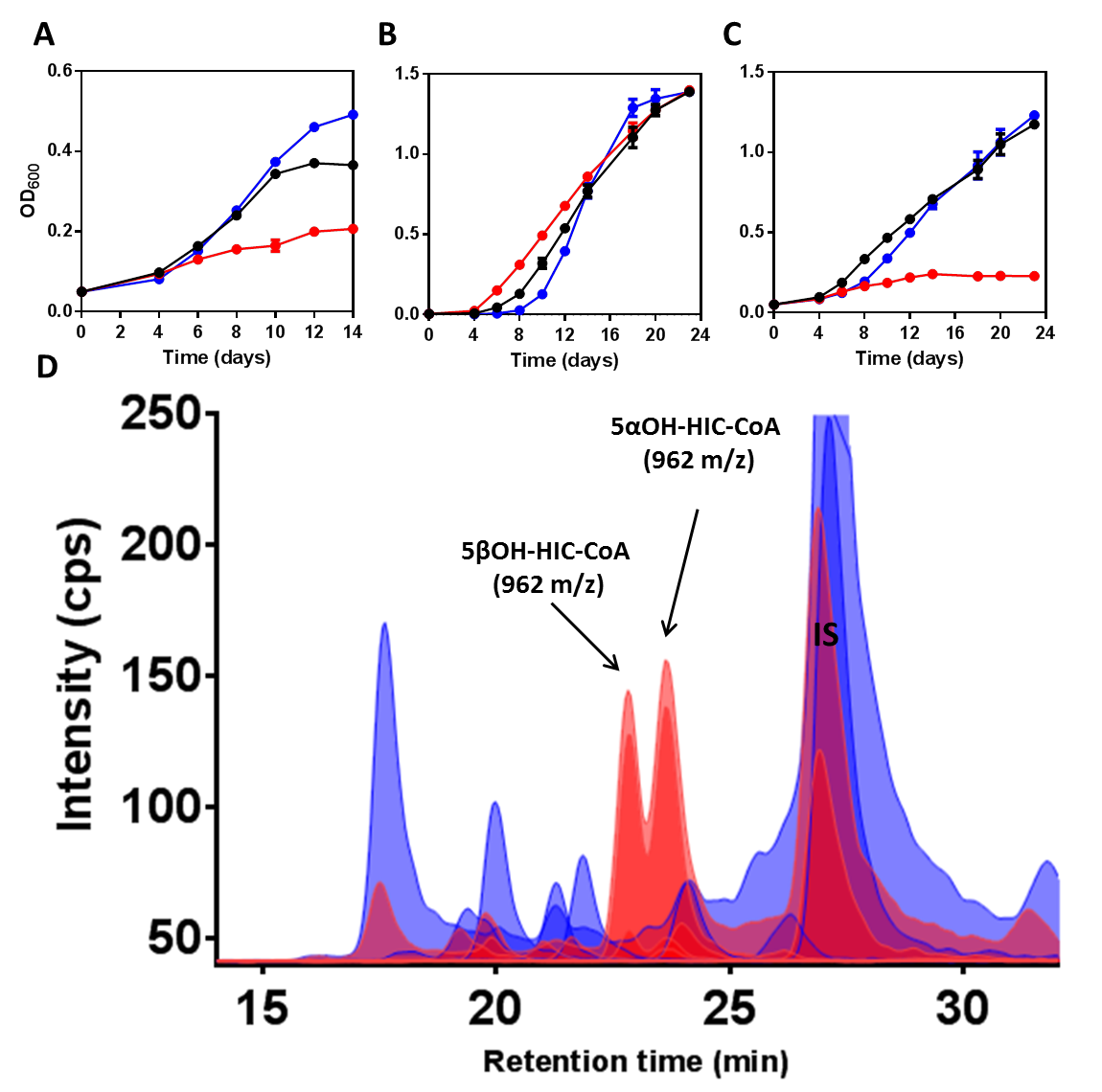

Supplement: FIG S2 [file mbo002173251sf2.tif]

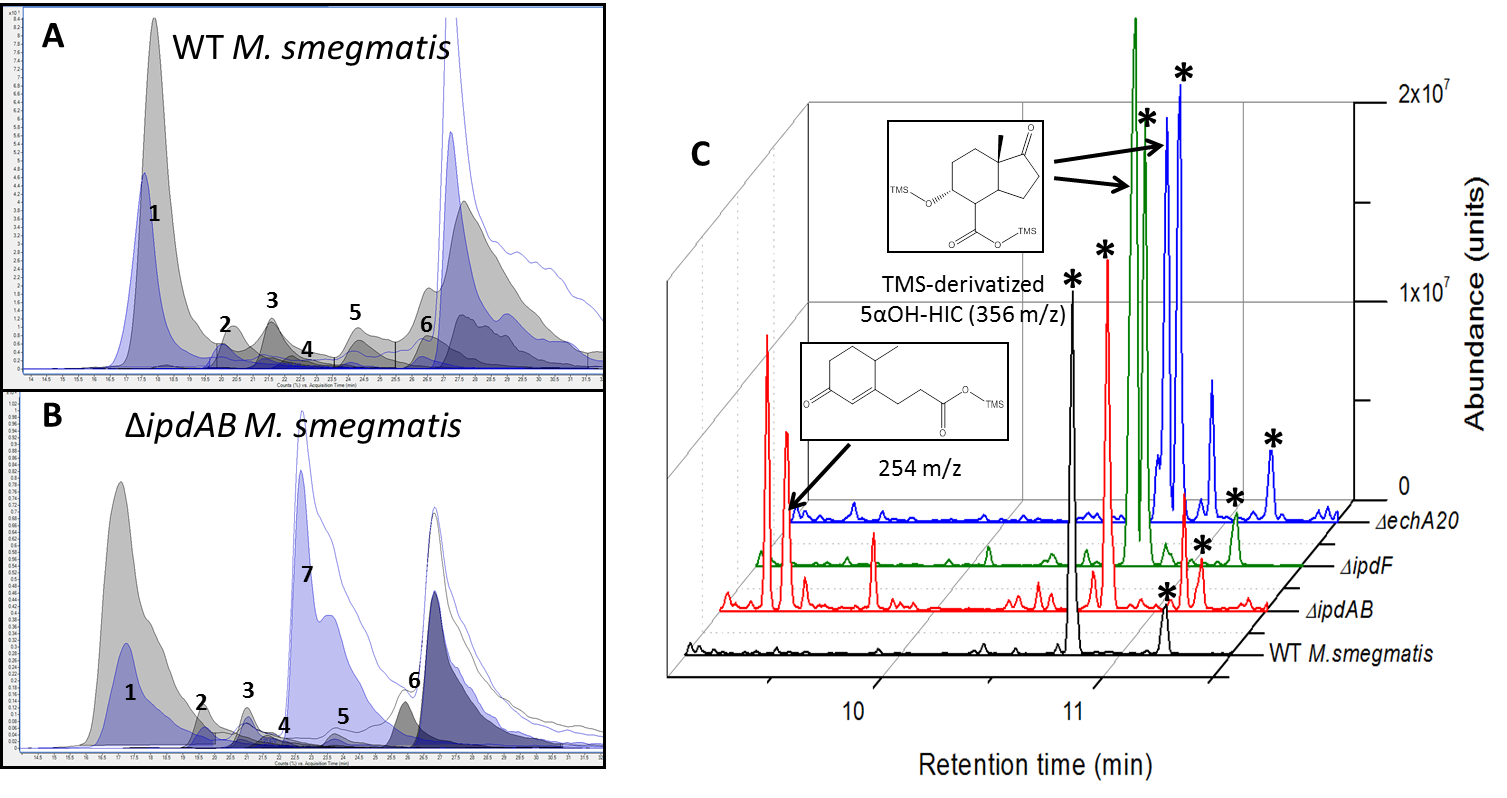

Supplement: FIG S3 [file mbo002173251sf3.tif]

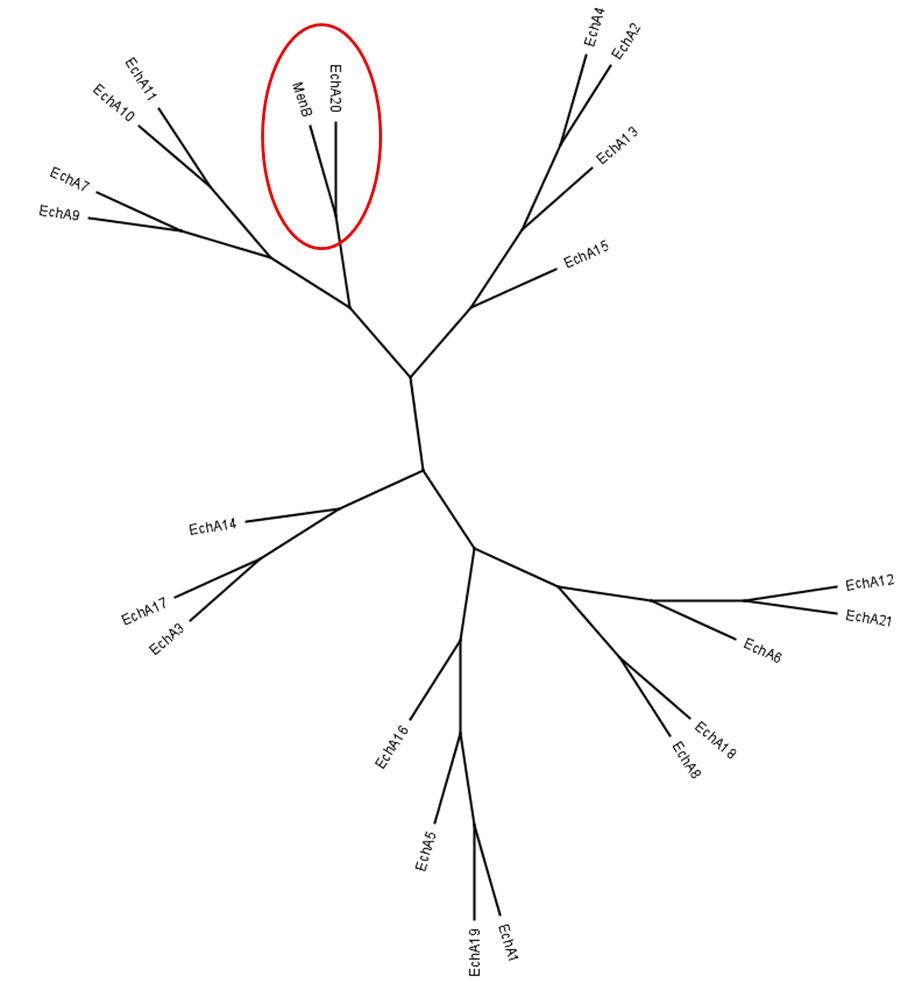

Supplement: FIG S4 [file mbo002173251sf4.tif]

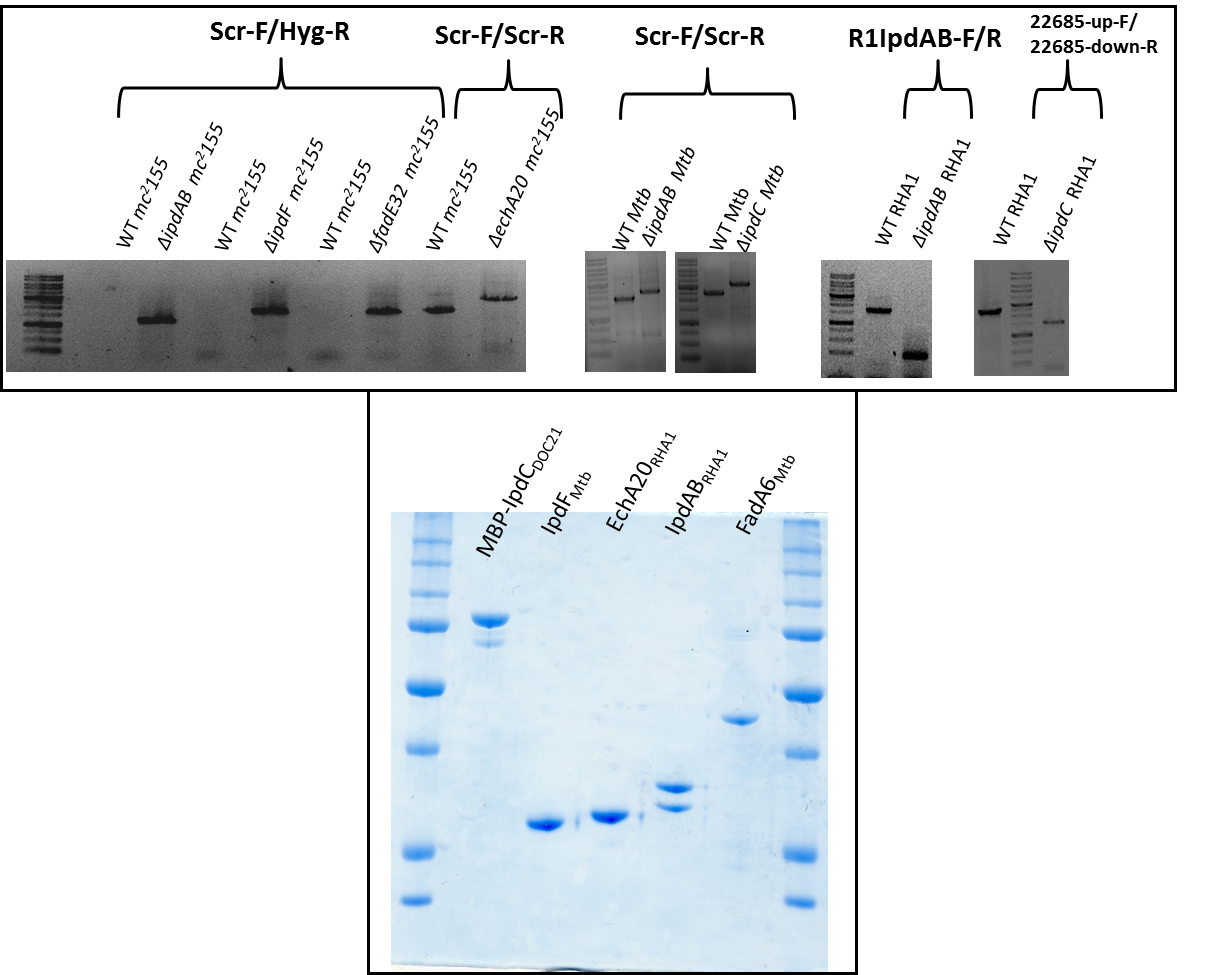

Supplement: FIG S5 [file mbo002173251sf5.tif]

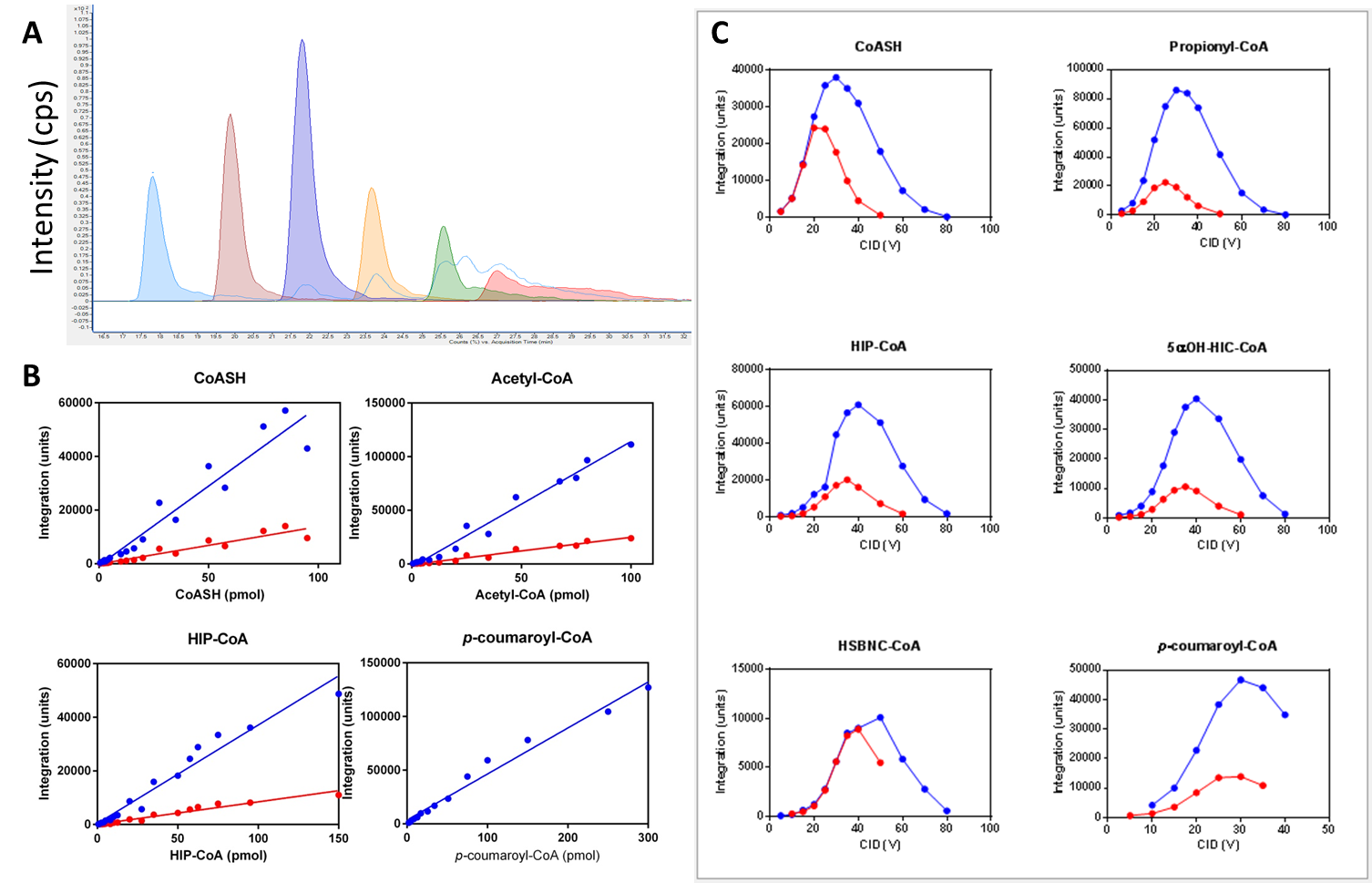

Supplement: FIG S6 [file mbo002173251sf6.tif]
