# Supplementary material for: Catabolism of the Last Two Steroid Rings in Mycobacterium tuberculosis and Other Bacteria
Source: mBio. 2017 Apr 4;8(2):e00321-17. doi: 10.1128/mBio.00321-17 (PMC5380842; doi:10.1128/mBio.00321-17)
Supplement: TABLE S1 [file mbo002173251st1.docx]

Table S1. Bacterial strains, constructs, and oligos used in this study.

| Name | Description | Reference |
| --- | --- | --- |
| **Strains** |  |  |
| RHA1 | *R. jostii* RHA1 | ([3](#_ENREF_3)) |
| Δ*ipdAB* RHA1 | RHA1 Δ*RS22695-22690* | This study |
| Δ*ipdC* RHA1 | RHA1 Δ*RS22685* | This study |
| Δ*ipdABC* RHA1 | RHA1 *ΔRS22685-22690* | This study |
| Δ*fadD3* RHA1 | RHA1 Δ*RS22410* | ([1](#_ENREF_1)) |
| *M. smegmatis* mc^2^155 |  | NCBI 246196 |
| Δ*echA20* mc^2^155 | mc^2^155 Δ*msmeg_6001*, Hyg^R^ | This study |
| Δ*echA20* mc^2^155*::echA20* | mc^2^155 Δ*msmeg_6001* containing pMVEchA20, Hyg^R^ Apr^R^ | This study |
| Δ*fadE32* mc^2^155 | mc^2^155 Δ*msmeg_6015*, Hyg^R^ | This study |
| Δ*fadE32 M* mc^2^155*::fadE32* | mc^2^155 Δ*msmeg_6015* containing pMVFadE32, Hyg^R^ Apr^R^ | This study |
| Δ*ipdF* mc^2^155 | mc^2^155 Δ*msmeg_6011*, Hyg^R^ | This study |
| Δ*ipdF M* mc^2^155*::rv3559c* | mc^2^155 Δ*msmeg_6011*containing pMVrv3559c, Hyg^R^ Apr^R^ | This study |
| Δ*ipdAB* mc^2^155 | mc^2^155 Δ*msmeg_6002-6003*, Hyg^R^ | This study |
| *Mtb* | *M. tuberculosis* Erdman | NCBI 652616 |
| Δ*ipdAB Mtb* | Erdman Δ*erdman_3896-3897*, Hyg^R^ | This study |
| Δ*ipdAB Mtb::ipdAB* | Erdman Δ*erdman_3896-3897* containing pMVipdAB, Hyg^R^ Apr^R^ | This study |
| Δ*ipdC Mtb* | Erdman Δ*erdman_3898*, Hyg^R^ | This study |
| Δ*ipdC Mtb::ipdABC* | Erdman Δ*erdman_3898* containing pMVipdABC, Hyg^R^ Apr^R^ | This study |
| *E. coli* DH5α |  | Invitrogen |
| *E. coli* Rosetta2 pLysS |  | Novagen |
| *E. coli* BL21(DE3) |  | NEB |
| **Plasmids** | | |
| pK18*mobsacB* | Plasmid for allelic exchange in RHA1. Kan^R^ | ([4](#_ENREF_4)) |
| pK18∆ipdAB | pK18*mobsacB* with upstream region of *RS22695* and downstream region of *RS22690* cloned into *Eco*RI/*Hin*dIII sites. Kan^R^ | This study |
| pK18∆ipdC | pK18*mobsacB* with upstream and downstream regions of *RS22685* cloned into *Eco*RI/*Hin*dIII sites. Kan^R^ |  |
| pYUB854 | Recombineering plasmid used for allelic exchange in mycobacteria*.* Hyg^R^ | ([5](#_ENREF_5)) |
| pYUBud6001 | pYUB854 containing upstream (*Afl*II/*Xba*I) and downstream (*Bgl*II/*Nhe*I) regions of *Msmeg*_*6001* flanking *hyg^R^*. Hyg^R^ | This study |
| pYUBud6011 | pYUB854 containing upstream (*Afl*II/*Xba*I) and downstream (*Bgl*II/*Nhe*I) regions of *Msmeg*_*6011,* flanking *hyg^R^*. Hyg^R^ | This study |
| pYUBud6015 | pYUB854 containing upstream (*Afl*II/*Xba*I) and downstream regions of *Msmeg*_*6015,* respectively, were introduced either side of *hyg^R^ Bgl*II/*Nhe*I (down). Hyg^R^ | This study |
| pYUBudipdAB | pYUB854 containing upstream (*Afl*II/*Xba*I) region of *Msmeg_6002* and downstream region of *Msmeg*_*6003* (*Bgl*II/*Nhe*I) flanking *hyg^R^* . Hyg^R^ | This study |
| pYUBudipdC | pYUB854 containing upstream (*Afl*II/*Xba*I) and downstream regions of *Msmeg*_*6004,* respectively, were introduced either side of *hyg^R^* into pYUB854 using AflII/XbaI (up) and *Bgl*II/*Nhe*I (down). Hyg^R^ | This study |
| pYUBudipdAB-mtb | pYUB854 containing upstream (*Afl*II/*Xba*I) region of *rv3551* and downstream region of *rv3552* (*Bgl*II/*Nhe*I) flanking *hyg^R^* . Hyg^R^ | This study |
| pYUBudipdC.mtb | pYUB854 containing upstream (*Afl*II/*Xba*I) region and downstream region of *rv3553* (*Bgl*II/*Nhe*I) flanking *hyg^R^* . Hyg^R^ | This study |
| pMV361.apr | Integrative plasmid with the constitutive *hsp60* promoter for complementation in mycobacteria. Apr^R^ | ([6](#_ENREF_6)) |
| pMVEchA20 | pMV361.apr containing *rv3550* inserted into the *Eco*RI/*Hind*III sites as a 766 bp fragment. A stop codon and RBS were added in the 5’UTR of the insert. Apr^R^ | This study |
| pMVFadE32 | pMV361.apr containing *msmeg_6015* inserted into the *Eco*RI/*Hind*III sites as a 988 bp fragment. A stop codon and RBS were added in the 5’UTR of the insert. Apr^R^ | This study |
| pMVrv3559c | pMV361.apr containing *rv3559c* inserted into the *Eco*RI/*Hind*III sites as a 811 bp fragment. A stop codon and RBS were added in the 5’UTR of the insert. Apr^R^ | This study |
| pMVipdAB | pMV361.apr containing *rv3551-3552c* inserted into the *Eco*RI/*Hind*III sites as a 1632 bp fragment. A stop codon and RBS were added in the 5’UTR of the transcript. Apr^R^ | This study |
| pMVechA20-ipdC | pMV361.apr containing *rv3550-3553c* inserted into the *Eco*RI/*Hind*III sites as a 3536 bp fragment. A stop codon and RBS were added in the 5’UTR of the insert. Apr^R^ | This study |
| pET41b+ | Expression vector for *E. coli*. Kan^R^ | Novagen |
| pETRv3559 | pET41b+ containing *rv3559c* (*ipdF*) with codons introducing an N-terminal His_6_ tag and TEV^Pro^ site following start codonas an 835 bp fragment inserted into *Nde*I/*Hind*III sites. Kan^R^ | This study |
| pTip-QC2 | Expression vector for RHA1. Thiostrepton inducible promoter. Cam^R^, Amp^R^ | ([7](#_ENREF_7)) |
| pTipR1EchA20 | pTip-QC2 Expression vector for EchA20_RHA1_*_._* 840 bp fragment containing *RS27700* (*echA20*) with codons introducing a hexahistidine tag and TEV^Pro^ site following start codon. as an 840 bp fragment inserted into *Eco*RI/*Hind*III sites. Cam^R^, Amp^R^ | This study |
| pTipFadA6 | pTip-QC2 containing *rv3556c* with codons introducing a hexahistidine tag and TEV^Pro^ site following start codon as a 1210 bp fragment inserted using *Eco*RI/*Hind*III into pTip-QC2. Cam^R^, Amp^R^ | This study |
| pTipR1IpdAB | pTip-QC2 containing *RS22695-22690* with codons introducing a hexahistidine tag and TEV^Pro^ site following start codon as a 1709 bp fragment inserted using *Eco*RI/*Hind*III into pTip-QC2. Cam^R^,Amp^R^ | This study |
| pTipIpdAB | pTip-QC2 containing *rv3551-2c* with codons introducing a hexahistidine tag and TEV^Pro^ site following start codon inserted using *Eco*RI/*Hind*III into pTip-QC2. Cam^R^,Amp^R^ | This study |
| pTipRv3553 | pTip-QC2containing *rv3553* inserted using *Eco*RI/*Hind*III into pTip-QC2. Cam^R^, Amp^R^ | This study |
| pMAL-c2x | Expression vector for *E.coli* used to make maltose binding protein (MBP)-fusion proteins. IPTG inducible promoter. Amp^R^ | (NEB #E8000S) |
| pMALDOC21 | Expression vector for MBP-IpdC_DOC21_ *_._*1116 bp fragment containing *DC0014_19.* Introduced into pMAL-c2x using *Eco*RI/*Hind*III adding *DC0014_19* in frame with MBP gene. Amp^R^ | This study |
| **Oligonucleotides** | | |

| Name | Use | Description | Sequence (5' – 3') |
| --- | --- | --- | --- |
| 22695Up-F | Deletion of *RHA1_RS22695-RS22690* to generate RHA1 Δ*ipdAB* | *Eco*RI site added at 5'; in combination with the 22695Up-R primer, generates an amplicon upstream of *RHA1_RS22695* with *Eco*RI and *Xba*I ends | CGAATTCGACCTGAATCCACGAATACCTCCG |
| 22695Up-R | Deletion of *RHA1_RS22695-RS22690* to generate RHA1 Δ*ipdAB* | *Eco*RI site added at 5'; in combination with the 22695Up-R primer, generates an amplicon upstream of *RHA1_RS22695* with *Eco*RI and *Xba*I ends | CTTCTAGAGATGCCGATGGTCATTCCGCTG |
| 22690Down-F | Deletion of *RHA1_RS22695-RS22690* to generate RHA1 Δ*ipdAB* | *Xba*I site added at 5'; in combination with the 22690Down-R primer, generates an amplicon downstream of *RHA1_RS22690* with *Xba*I and *Hin*dIII ends | CTTCTAGAGAACCCACCGACGAAGAACTG |
| 22690Down-R | Deletion of *RHA1_RS22695-RS22690* to generate RHA1 Δ*ipdAB* | *Hin*dIII site added at 5'; in combination with the 22690Down-F primer, generates an amplicon downstream of *RHA1_RS22690* with *Xba*I and *Hin*dIII ends | GCTAAGCTTCGAGGTATTCGTGTTTGACGG |
| 22685Up-F | Deletion of *RHA1_RS22685* to generate RHA Δ*ipdC* | *Eco*RI site added at 5'; in combination with the 22685Up-R primer, generates an amplicon upstream of *RHA1_RS22685* with *Eco*RI and *Xba*I ends | CCTAGAATTCCCGACCTGCTGATCACCGACGG |
| 22685Up-R | Deletion of *RHA1_RS22685* to generate RHA Δ*ipdC* | *Xba*I site added at 5'; in combination with the 22685Up-F primer, generates an amplicon upstream of *RHA1_RS22685* with *Eco*RI and *Xba*I ends | ACCTCTAGACTGGACCACCGGGTGCTGCAC |
| 22685Down-F | Deletion of *RHA1_RS22685* to generate RHA Δ*ipdC* | *Xba*I site added at 5'; in combination with the 22685Down-R primer, generates an amplicon downstream of *RHA1_RS22685* with *Xba*I and *Hin*dIII ends | AGATCTAGACTGCTCGACGACCTGCCCACATG |
| 22685Down-R | Deletion of *RHA1_RS22685* to generate RHA Δ*ipdC* | *Hin*dIII site added at 5'; in combination with the 22685Down-F primer, generates an amplicon downstream of *RHA1_RS22685* with *Xba*I and *Hin*dIII ends | GCTAAGCTTCTGATCGCACCGCAGTACCGCAG |
| 22685-22695Up-F | Deletion of *RHA1_RS22685-RS22695* to generate RHA Δ*ipdABC* | *Eco*RI site added at 5'; in combination with the 22685-22695Up-R primer, generates an amplicon upstream of *RHA1_RS22695* with *Eco*RI and *Bam*HI ends | CCTAGAATTCCGACGGAGACGGCATCACCACG |
| 22685-22695Up-R | Deletion of *RHA1_RS22685-RS22695* to generate RHA Δ*ipdABC* | *Bam*HI site added at 5'; in combination with the 22685-22695Up-F primer, generates an amplicon upstream of *RHA1_RS22695* with *Eco*RI and *Bam*HI ends | AGAGGATCCCTGGACCACCGGGTGCTGCAC |
| 22685-22695Down-F | Deletion of *RHA1_RS22685-RS22695* to generate RHA Δ*ipdABC* | *Bam*HI site added at 5'; in combination with the 22685-22695Down-R primer, generates an amplicon downstream of *RHA1_RS22685* with *Bam*HI and *Hin*dIII ends | AGAGGATCCCTGCTCGACGACCTGCCCACATG |
| 22685-22695Down-R | Deletion of *RHA1_RS22685-RS22695* to generate RHA Δ*ipdABC* | *Hin*dII site added at 5'; in combination with the 22685-22695Down-F primer, generates an amplicon downstream of *RHA1_RS22685* with *Bam*HI and *Hin*dIII ends | GCTAAGCTTCTGATCGCACCGCAGTACCGCAG |
| 22410Up-F | Deletion of *RHA1_RS22410* to generate RHA Δ*fadD3* | *Kpn*I site added at 5'; in combination with the 22410Up-R primer, generates an amplicon upstream of *RHA1_RS22410* with *Kpn*I and *Xba*I ends | TATGGTACCGTCGCCCAGTTCGAGATGTTCG |
| 22410Up-R | Deletion of *RHA1_RS22410* to generate RHA Δ*fadD3* | *Xba*I site added at 5'; in combination with the 22410Up-F primer, generates an amplicon upstream of *RHA1_RS22410* with *Kpn*I and *Xba*I ends | GGTTCTAGATCCGCCACGGCTGTGAGCTC |
| 22410Down-F | Deletion of *RHA1_RS22410* to generate RHA Δ*fadD3* | *Xba*I site added at 5'; in combination with the 22410Down-R primer, generates an amplicon downstream of *RHA1_RS22410* with *Bam*HI and *Hin*dIII ends | CTCTCTAGAATCGGTGCGCTTCGTCGACTC |
| 22410Down-R | Deletion of *RHA1_RS22410* to generate RHA Δ*fadD3* | *Hin*dIII site added at 5'; in combination with the 22410Down-F primer, generates an amplicon downstream of *RHA1_RS22410* with *Bam*HI and *Hin*dIII ends | AATAAGCTTCACCTCCTCGCGGAAGGCG |
| Rv3551up-F | Deletion of *rv3551-3552* to generate *Mtb ΔipdAB* | *Afl*II site added at 5'; in combination with the Rv3551up-R primer, generates an amplicon upstream of *rv3551* with *Afl*II and *Xba*I ends | TGTCTTAAGTAGGTAGCGAACCCGCAGGAGTGC |
| Rv3551up-R | Deletion of *rv3551-3552* to generate *Mtb ΔipdAB* | *Xba*I site added at 5'; in combination with the Rv3551up-F primer, generates an amplicon upstream of *rv3551* with *Afl*II and *Xba*I ends | GATCTAGAGGTTCGTTTATCGGGCACTATGACTTC |
| Rv3551down-F | Deletion of *rv3551-3552* to generate *Mtb ΔipdAB* | *Xho*I site added at 5'; in combination with the Rv3551down-R primer, generates an amplicon downstream of *rv3552* with *Xho*I and *Bgl*II ends | CCTCTCGAGCACCGACGACGAACTGCACCTG |
| Rv3551down-R | Deletion of *rv3551-3552* to generate *Mtb ΔipdAB* | *Bgl*II site added at 5'; in combination with the Rv3551down-F primer, generates an amplicon downstream of *rv3552* with *Xho*I and *Bgl*II ends | GTAGATCTCGGCAACAGCAGCGTGGTGG |
| Rv3553up-F | Deletion of *rv3553* to generate *Mtb ΔipdC* | *Afl*II site added at 5'; in combination with the Rv3553up-R primer, generates an amplicon upstream of *rv3553* with *Afl*II and *Xba*I ends | TTACTTAAGGAGGCTCAGCTGCTCGCGGACAC |
| Rv3553up-R | Deletion of *rv3553* to generate *Mtb ΔipdC* | *Xba*I site added at 5'; in combination with the Rv3553up-F primer, generates an amplicon upstream of *rv3553* with *Afl*II and *Xba*I ends | GGTCTAGATCTGCACCACCGGGTGCTCGATG |
| Rv3553down-F | Deletion of *rv3553* to generate *Mtb ΔipdC* | *Nhe*I site added at 5'; in combination with the Rv3553down-R primer, generates an amplicon downstream of *rv3553* with *Nhe*I and *Bgl*II ends | ACTGCTAGCGCATTCTTGACGACCTACCGTCGTG |
| Rv3553down-R | Deletion of *rv3553* to generate *Mtb ΔipdC* | *Bgl*II site added at 5'; in combination with the Rv3553down-F primer, generates an amplicon downstream of *rv3553* with *Xho*I and *Bgl*II ends | GTAGATCTCAGGGTCATCATCACCAGCGTCTCG |
| 6002Up-F | Deletion of *MSMEG_6002-6003* to generate *M. smegmatis ΔipdAB* | *Afl*II site added at 5'; in combination with the 6002Up-R primer, generates an amplicon upstream of *MSMEG_6002* with *Afl*II and *Xba*I ends | TTACTTAAGGAACTCGCAGACGCGATCACCG |
| 6002Up-R | Deletion of *MSMEG_6002-6003* to generate *M. smegmatis ΔipdAB* | *Xba*I site added at 5'; in combination with the 6002Up-F primer, generates an amplicon upstream of *MSMEG_6002* with *Afl*II and *Xba*I ends | GGTCTAGAGCCGATGGTCATGCCGCTCTC |
| 6003Down-F | Deletion of *MSMEG_6002-6003* to generate *M. smegmatis ΔipdAB* | *Nhe*I site added at 5'; in combination with the 6003down-R primer, generates an amplicon downstream of *MSMEG_6003* with *Nhe*I and *Bgl*II ends | CCTGCTAGCCAGCGAGGACGAGCTCAAGCTG |
| 6003Down-R | Deletion of *MSMEG_6002-6003* to generate *M. smegmatis ΔipdAB* | *Bgl*II site added at 5'; in combination with the 6003down-F primer, generates an amplicon downstream of *MSMEG_6003* with *Nhe*I and *Bgl*II ends | GTAGATCTCAGACCACGCCCGTCGAAGAATC |
| 6011Up-F | Deletion of *MSMEG_6011* to generate *M. smegmatis ΔipdF* | *Afl*II site added at 5'; in combination with the 6011Up-R primer, generates an amplicon upstream of *MSMEG_6011* with *Afl*II and *Xba*I ends | TTACTTAAGATGGGCACGCTGACCTTCGAG |
| 6011Up-R | Deletion of *MSMEG_6011* to generate *M. smegmatis ΔipdF* | *Afl*II site added at 5'; in combination with the 6011Up-F primer, generates an amplicon upstream of *MSMEG_6011* with *Afl*II and *Xba*I ends | GGTCTAGAGTCGGAGATCACCACGTCGGC |
| 6011Down-F | Deletion of *MSMEG_6011* to generate *M. smegmatis ΔipdF* | *Nhe*I site added at 5'; in combination with the 6011down-R primer, generates an amplicon downstream of *MSMEG_6011* with *Nhe*I and *Bgl*II ends | CCTGCTAGCTACGTCGTCCTCGGAACTGCTGG |
| 6011Down-R | Deletion of *MSMEG_6011* to generate *M. smegmatis ΔipdF* | *Bgl*II site added at 5'; in combination with the 6011Down-F primer, generates an amplicon downstream of *MSMEG_6011* with *Nhe*I and *Bgl*II ends | GTAGATCTCGCATCGGCATTTGTTCTCGC |
| 6001Up-F | Deletion of *MSMEG_6001* to generate *M. smegmatis ΔechA20* | *Afl*II site added at 5'; in combination with the 6011Up-R primer, generates an amplicon upstream of *MSMEG_6001* with *Afl*II and *Xba*I ends | TTACTTAAGCGCTGACGCTGGAGATGTTGACG |
| 6001Up-R | Deletion of *MSMEG_6001* to generate *M. smegmatisΔechA20* | *Afl*II site added at 5'; in combination with the 6011Up-F primer, generates an amplicon upstream of *MSMEG_6001* with *Afl*II and *Xba*I ends | GGTCTAGAGAACCAGCCACGCGACGGAAC |
| 6001Down-F | Deletion of *MSMEG_6001* to generate *M. smegmatis ΔechA20* | *Nhe*I site added at 5'; in combination with the 6011down-R primer, generates an amplicon downstream of *MSMEG_6001* with *Nhe*I and *Bgl*II ends | ACTGCTAGCCATGGAACAGGGCTTCACGTTCG |
| 6001Down-R | Deletion of *MSMEG_6001* to generate *M. smegmatis ΔechA20* | *Bgl*II site added at 5'; in combination with the 6011Down-F primer, generates an amplicon downstream of *MSMEG_6001* with *Nhe*I and *Bgl*II ends | GTAGATCTCGTCACCGAGATTCATGTGCACG |
| 6015Up-F | Deletion of *MSMEG_6015* to generate *M. smegmatis ΔfadE32* | *Afl*II site added at 5'; in combination with the 6011Up-R primer, generates an amplicon upstream of *MSMEG_6015* with *Afl*II and *Xba*I ends | TTACTTAAGCGATGAGCACGTCGAGCAACGAG |
| 6015Up-R | Deletion of *MSMEG_6015* to generate *M. smegmatis ΔfadE32* | *Afl*II site added at 5'; in combination with the 6011Up-F primer, generates an amplicon upstream of *MSMEG_6015* with *Afl*II and *Xba*I ends | GGTCTAGAAGCTGTGCCCAGACCTTGCGG |
| 6015Down-F | Deletion of *MSMEG_6015* to generate *M. smegmatis ΔfadE32* | *Nhe*I site added at 5'; in combination with the 6011down-R primer, generates an amplicon downstream of *MSMEG_6015* with *Nhe*I and *Bgl*II ends | ACTGCTAGCACGACCTGTCGTTGTTGTTGCTGC |
| 6015Down-R | Deletion of *MSMEG_6015* to generate *M. smegmatis ΔfadE32* | *Bgl*II site added at 5'; in combination with the 6011Down-F primer, generates an amplicon downstream of *MSMEG_6015* with *Nhe*I and *Bgl*II ends | GTAGATCTCGACGGTCAGGTCCAGGCATCG |
| pMV361ipdAB-F | Amplification of *rv3551-3552* to complement *Mtb* Δ*ipdAB* | *Eco*RI and ribosome binding site (RBS) added to 5'. Adds stop codon TAA in front of the RBS to stop the potential expression of the fusion protein from pMV361. | ACGGAATTCTAAAAGGAGATCACTCATGCCCGATAAACGAACCGCTCTTG |
| pMV361ipdAB-R | Amplification of *rv3551-3552* to complement *Mtb* Δ*ipdAB* | *Hin*dIII cut site added at 5'. In combination with pMV361ipdAB-F primer, generates an amplicon from *rv3551* to 62 bp downstream of *rv3552* with *Eco*RI and *Hin*dIII ends. | TTTAAGCTTCTTGCAGGGGACGAAGCGATG |
| pMV361rv3550-3553-F | Amplification of *rv3550-3553* to complement *Mtb* Δ*ipdC* | *Eco*RI and ribosome binding site (RBS) added to 5'. Adds stop codon TAA in front of the RBS to stop the potential expression of the fusion protein from pMV361. | ACGGAATTCTAAAAGGAGATCACTCATGCCGATCACCTCCA |
| pMV361rv3550-3553-R | Amplification of *rv3550-3553* to complement *Mtb* Δ*ipdC* | *Hin*dIII cut site added at 5'. In combination with pMV361rv3550-3553-F primer, generates an amplicon from *rv3550* to 181 bp downstream of *rv3553* with *Eco*RI and *Hin*dIII ends. | TTTAAGCTTGTCGTAGAACCGACGA |
| compEchA20-F | Amplification of *rv3550* to complement *M. smegmatis* Δ*echA20* | *Eco*RI and ribosome binding site (RBS) added to 5'. Adds stop codon TAA in front of the RBS to stop the potential expression of the fusion protein from pMV361. | ACGGAATTCTAAAAGGAGATCACTCATGCCGATCACCTCCACCACG |
| compEchA20-R | Amplification of *rv3550* to complement *M. smegmatis* Δ*echA20* | *Hin*dIII cut site added at 5'. In combination with compEchA20-F primer, generates an amplicon of *rv3550* with *Eco*RI and *Hin*dIII ends. | CGCTTCGAACTATGACTTCTTCACAAAGGCGTCGC |
| compFadE32-F | Amplification of *MSMEG_6015* to complement *M. smegmatis* Δ*fadE32* | *Eco*RI and ribosome binding site (RBS) added to 5'. Adds stop codon TAA in front of the RBS to stop the potential expression of the fusion protein from pMV361. | ACGGAATTCTAAAAGGAGATCACTCATGAACTTCGAAACCGACGAA |
| compFadE32-R | Amplification of *MSMEG_6015* to complement *M. smegmatis* Δ*fadE32* | *Hin*dIII cut site added at 5'. In combination with compFadE32-F primer, generates an amplicon of *MSMEG_6015* with *Eco*RI and *Hin*dIII ends. | CGCTTCGAATCACGACAGGCCCTCCAGT |
| compIpdF-F | Amplification of *rv3559* to complement *M. smegmatis* Δ*ipdF* | *Eco*RI and ribosome binding site (RBS) added to 5'. Adds stop codon TAA in front of the RBS to stop the potential expression of the fusion protein from pMV361. | ACGGAATTCTAAAAGGAGATCACTCATGAACCTGTCCGTAGCGCCG |
| compIpdF-R | Amplification of *rv3559* to complement *M. smegmatis* Δ*ipdF* | *Hin*dIII cut site added at 5'. In combination with compIpdF-F primer, generates an amplicon of *rv3559* with *Eco*RI and *Hin*dIII ends. | CGGTTCGAATCACGGGTGCTGGCAGGATA |
| R1EchA20-F | Amplification of *RHA1_RS27700* to make pTipR1EchA20 | *Nde*I site added at 5'; 6x Histidine tag and thrombin cleavage site added at 5'; in combination with echA20-R, generates an amplicon containing *RHA1­_RS277700* with *Nde*1 and *Hin*dIII ends | GCAATATCATATGCATCACCATCACCATCACGAGAACCTGTACTTCCAGTCGGGCATCACCTCCACCACCGAC |
| R1EchA20-R | Amplification of *RHA1_RS27700* to make pTipR1EchA20 | *Hin*dIII site added at 5'; in combination with echA20-F, generates an amplicon containing *RHA1­_RS277700* with *Nde*1 and *Hin*dIII ends | CGGAAGCTTGCTTGCTCACCATCGATGTCAGCC |
| FadA6-F | Amplification of *rv3556c* to make pTipFadA6 | *Nde*I site added at 5'; 6x Histidine tag and thrombin cleavage site added at 5'; in combination with fadA6-R, generates an amplicon containing *rv3556c* with *Nde*1 and *Hin*dIII ends | GCAATAGCATATGCATCACCATCACCATCACGAGAACCTGTACTTCCAGTCGACAGAGGCGTACGTCATCGACG |
| FadA6-R | Amplification of *rv3556c* to make pTipFadA6 | *Hin*dIII site added at 5'; in combination with fadA6-F, generates an amplicon containing *rv3556c* with *Nde*1 and *Hin*dIII ends | CGTAAGCTTAACGAAGGCCTACAGCCGCTC |
| R1IpdAB-F | Amplification of *RHA1_RS22695-RS22690* to make pTipR1IpdAB | *Nde*I site added at 5'; 6x Histidine tag and thrombin cleavage site added at 5'; in combination with R1IpdAB-R, generates amplicon containing *RHA1_RS22695-RS22690 with Nde*1 and *Hin*dIII ends | GCAATAGCATATGCATCACCATCACCATCACCTCGTCCCGCGCGGCTCGGTGAGCAAGCGCGACAAGAG |
| R1IpdAB-R | Amplification of *RHA1_RS22695-RS22690* to make pTipR1IpdAB | *Hin*dIII site added at 5'; in combination with R1IpdAB-F, generates amplicon containing *RHA1_RS22695-RS22690 with Nde*1 and *Hin*dIII ends | CGTAAGCTTCGGTGCGGAGCGTGCTCATAC |
| IpdAB-F | Amplification of *Rv3551-3552* to make pTipIpdAB | *NdeI* site added at 5'; in combination with IpdAB-R, generates amplicon containing *Rv3551-3552 with Nde*1 and *Hin*dIII ends | GCTGAACCATATGCCCGATAAACGAACCGCTC |
| IpdAB-R | Amplification of *Rv3551-3552* to make pTipIpdAB | *Hin*dIII site added at 5'; in combination with IpdAB-F, generates amplicon containing *Rv3551-3552 with Nde*1 and *Hin*dIII ends | CGTAAGCTTGGGAGAGGAGGCGGAACAATC |
| Rv3559c-F | Amplification of *rv3559c* to make pETRv3559c | *Nde*I site added at 5'; 6x Histidine tag and thrombin cleavage site added at 5'; in combination with Rv3559-R, generates amplicon containing *rv3559c with Nde*1 and *Hin*dIII ends | GCAATATCATATGCATCACCATCACCATCACGAGAACCTGTACTTCCAGTCGAACCTGTCCGTAGCGCCGA |
| Rv3559c-R | Amplification of *rv3559c* to make pETRv3559c | *Hin*dIII site added at 5'; in combination with Rv3559-F, generates amplicon containing *rv3559c with Nde*1 and *Hin*dIII ends | CGCAAGCTTCTCACGGGTGCTGGCAGGAT |
| Rv3553-F | Amplification of *Rv3553* to make pTipRv3553 | *Nde*I site added at 5'; 6x Histidine tag and thrombin cleavage site added at 5'; in combination with Rv3553-R, generates amplicon containing *Rv3553 with Nde*1 and *Hin*dIII ends | GCTGAACCATATGGCTACTAGTCATCACCATCACCATCACGGCAGCTCTGAGAACCTGTACTTCCAGTCGAGGCTGCGTACGCCGCTG |
| Rv3553-R | Amplification of *Rv3553* to make pTipRv3553 | *NdeI* site added at 5'; in combination with Rv3553-R, generates amplicon containing *Rv3553 with Nde*1 and *Hin*dIII ends | GTCAAGCTTTCACTCCACCAGCGCAGATGC |
| DOC21-F | Amplification of *DOC0014_19* to make pMALDOC21 | *EcoRI* site added at 5’, in combination with DOC21-R generates amplicon containing *DOC0014_19* with *EcoR1* and *HindIII* ends | CACGAATTCATGAGCGGCTGGCTGAACACTCC |
| DOC21-R | Amplification of *DOC0014_19* to make pMALDOC21 | *HindIII* site added at 5’, in combination with DOC21-F generates amplicon containing *DOC0014_19* with *EcoR1* and *HindIII* ends | CGGAAGCTTTCACAGGGGTTCTCCAATAGGGG |
| ipdC-scn-F | Screening of *Mtb ΔipdC* | Binds *rv3553c* 102 bp upstream of Rv3553-Up-F primer | CGATGCAGGCGAAATCATGATCAGC |
| ipdC-scn-R | Screening of *Mtb ΔipdC* | Binds *rv3553c* 95 bp downstream of Rv3553-Dn-R primer | CAGCACGGTAAGGCCGATACGTTG |
| 6002-Scr-F | Screening of *M. smegmatis ΔipdAB* | Binds *MSMEG_6002* 89 bp upstream of 6002-Up-F primer | CGATCACATCCACCACCGTCGAAC |
| 6003-Scr-R | Screening of *M. smegmatis ΔipdAB* | Binds *MSMEG_6003* 58 bp downstream of 6003-Dn-R primer | CGGTGCGGCATACCGTCCACAC |
| Scr-EchA20-F | Screening of *M. smegmatis ΔechA20* | Binds upstream of *MSMEG_6001* | CCATTCGACGGCCAGGGTCTC |
| Scr-EchA20-R | Screening of *M. smegmatis ΔechA20* | Binds downstream of *MSMEG_6001* | CACGGCCTTGACCAGTTCGTCG |
| Scr-6011-F | Screening of *M. smegmatis ΔipdF* | Binds 150 bp upstream of *MSMEG_60011* | CTGTTGGTGCCGCTCGATCAG |
| Scr-6011-R | Screening of *M. smegmatis ΔipdF* | Binds 196 bp downstream of *MSMEG_6011* | CCAGCGTCTCGTCATCGGGTG |
| Scr-FadE32-F | Screening of *M. smegmatis ΔfadE32* | Binds upstream of *MSMEG_6015* | GCCTGACCTACTTCATGTTCGACC |
| Scr-FadE32-R | Screening of *M. smegmatis ΔfadE32* | Binds upstream of *MSMEG_6015* | CCGAGGCGACCTTGACGTACAG |
| Hyg-R | Screening deletions in *M.smegmatis* | Binds internally to *hyg^R^* near 3’ end | GTCATCCGGCTCATCACCAGGTAG |
