# Supplementary material for: Catabolism of the Last Two Steroid Rings in Mycobacterium tuberculosis and Other Bacteria
Source: mBio. 2017 Apr 4;8(2):e00321-17. doi: 10.1128/mBio.00321-17 (PMC5380842; doi:10.1128/mBio.00321-17)
Supplement: TABLE S2 [file mbo002173251st2.docx]

**Table S2. Characterization of CoA metabolites found in this study.**

| Compound | Full Name | M+H (QQQ)^a^ | MRM Transition | M+H (Bruker)^b^ | Molecular Formula | Retention time (min) | NMR Confirmed | Reference | Major peak observed in CoA metabolome^c^ | *in vitro* enzymatic production^d^ |
| --- | --- | --- | --- | --- | --- | --- | --- | --- | --- | --- |
| CoASH | Coenzyme-A | 768.1 | 768=> 261,428 | 768.1210 | C_21_H_37_N_7_O_16_P_3_S^+^ | 17.0-18.4 | Yes | SIGMA | All |  |
| Dephos-CoASH | Dephospho-CoASH | 688.1 | 688=>348 | 688.1562 | C_21_H_36_N_7_O_13_P_2_S^+^ | 19.3 | No |  | All |  |
| Acetyl-CoA |  | 810.1 | 810=> 303,428 | 810.1315 | C_23_H_39_N_7_O_17_P_3_S^+^ | 19.9-20.9 | yes | SIGMA | All | COCHEA-CoA + IpdAB_RHA1_ + FadA6_Mtb_ |
| Propionyl-CoA |  | 824.1 | 824=> 317,428 | 824.1466 | C_24_H_41_N_7_O_17_P_3_S^+^ | 21.6-22.9 | Yes |  | All |  |
| Succinyl-CoA |  | 868.1 | 868=> 361,428 | 868.1400 | C_25_H_41_N_7_O_19_P_3_S^+^ | 21.6-22.2 | Yes |  | All |  |
| Unknown | Unknown | 992.2 | 992=> 485,428 | 992.1901 | C_32_H_49_N_7_O_21_P_3_S^+^ | 20.8-22.0 | No |  | RHA1 *ΔipdAB*  *Mtb ΔipdAB Δ MC^2^155 ΔipdAB* |  |
| 5β-OH HIC-CoA | 3aβ-*H*-4*α*(Carboxylic acid)-5*α*-hydroxy-7a*β*-methylhexahydro-1-indanone | 962.1 | 962=> 455,428 | 962.2159 | C_32_H_51_N_7_O_19_P_3_S^+^ | 22.0-22.9 | Yes (5βOH-HIC) | This Study | RHA1 *ΔipdC, ΔipdABC Mtb ΔipdC* |  |
| HIEC-CoA | (7aS)-7a-Methyl-1,5-dioxo-2,3,5,6,7,7a-hexahydro-1H-indene-4-carboxyl-CoA | 958.1 | 958=> 451,428 | 958.1899 | C_32_H_47_N_7_O_19_P_3_S^+^ | 23.2-23.4 | No | This Study | *MC^2^155 ΔechA20* | 5α-OH HIC-CoA + IpdC_DOC21_+IpdF_Mtb_ |
| COCHEA-CoA | (3R)-2-(2-Carboxyethyl)-3-methyl-6-oxocyclohex-1-ene-1-carboxyl-CoA | 976.2 | 976=> 469,428 | 976.1920 | C_32_H_49_N_7_O_20_P_3_S^+^ | 22.7-23.4 | Yes | This Study | RHA1 Δ*ipdAB*  *MC^2^155* Δ*ipdAB*  *Mtb* Δ*ipdAB* | HIEC-CoA + EchA20_RHA1_ |
| MOODA-CoA | 4-Methyl-5-oxooctanedioyl-CoA | 952.2 | 952=> 445,428 | 952.1960 | C_30_H_49_N_7_O_20_P_3_S^+^ | 23.0-23.2 | Yes (MOODA) | This Study | *MC^2^155* *ΔfadE32* (MOODA in Sup.) | COCHEA-CoA + IpdAB_RHA1_ + FadA6_Mtb_ |
| 5α-OH HIC-CoA | 3a*α*-*H*-4*α*(Carboxylic acid)-5*α*-hydroxy-7a*β*-methylhexahydro-1-indanone | 962.1 | 962=> 455,428 | 962.2159 | C_32_H_51_N_7_O_19_P_3_S^+^ | 23.0-23.8 | Yes (5αOH-HIC) | This Study | RHA1 *ΔipdC,* *ΔipdABC*  *Mtb ΔipdC*  *MC^2^155 ΔechA20, ΔipdF* |  |
| 3'oxo-5OH-HIP-CoA |  | 1004.2 | 1004=> 453,428 | 1004.2265 | C_34_H_53_N_7_O_20_P_3_S^+^ | 23.3-24.1 | No | This Study |  |  |
| 3',5-diOH-HIP-CoA |  | 1006.2 | 1006=> 499,428 | 1006.2420 | C_34_H_55_N_7_O_20_P_3_S^+^ | 24.0-24.2 | No | This Study |  |  |
