# Supplementary material for: Catabolism of the Last Two Steroid Rings in Mycobacterium tuberculosis and Other Bacteria
Source: mBio. 2017 Apr 4;8(2):e00321-17. doi: 10.1128/mBio.00321-17 (PMC5380842; doi:10.1128/mBio.00321-17)
Supplement: TEXT S1 [file mbo002173251s1.docx]

**Supplemental Information for Catabolism of the last two cholesterol rings in *Mycobacterium tuberculosis* and other bacteria.**

Adam M. Crowe^£,*^, Israël Casabon^ǂ,*^, Kirstin L. Brown^ǂ^, Jie Liu^ǂ^, Jennifer Lian^ǂ^, Jason C. Rogalski^γ^, Timothy E. Hurst^ξ^, Victor Snieckus^ξ^, Leonard J. Foster^γ^ and Lindsay D. Eltis^ǂ,£,¶^

^¶^E-mail: [leltis@mail.ubc.ca](mailto:leltis@mail.ubc.ca)

**MATERIALS AND METHODS**

*Chemicals and Reagents.* ATP, *p*-coumaric acid, propionic anhydride, succinic anhydride, CoASH, phorbol 12-myristate 13-acetate (PMA), sodium acetate, sodium propionate, and cholesterol (>99%) were purchased from Sigma-Aldrich. Restriction enzymes and T4 DNA ligase were purchased from New England Biolabs. ACCUZYME was purchased from Bioline. All other reagents were of HPLC or analytical grade. Buffers and solvents were prepared as previously described ([1](#_ENREF_1)).

*Growth of bacteria.* Strains and plasmids used in this study are provided below (Table S1). RHA1 strains were cultivated aerobically at 30°C on M9 mineral medium as described previously ([1](#_ENREF_1)) containing either 1 mM cholesterol, 1.5 mM HIP, or 10 mM pyruvate. *M. smegmatis* strains were cultivated aerobically at 37°C in M9 supplemented with 2 mM MgSO_4_ and 0.1 mM CaCl_2_ as described previously ([2](#_ENREF_2)). *Mtb* strains were grown on 7H9 media supplemented with 0.5% tyloxapol and either 0.2% glycerol or 0.5 mM cholesterol as described previously ([3](#_ENREF_3)). *Escherichia coli* strains were grown at 37°C in Luria-Bertani (LB) broth. Hygromycin (150 µg ml^-1^ for *E. coli*, 50 µg ml^-1^ for mycobacteria), kanamycin (25 µg ml^-1^ for *E. coli*, 20 µg ml^-1^ for mycobacteria), ampicillin (100 µg ml^-1^), chloramphenicol (34 µg ml^-1^) and apramycin (30 µg ml^-1^) were used for selection where appropriate. Growth of RHA1 and *M. smegmatis* was followed using OD_600 nm_. Growth of *Mtb* was followed using OD_600 nm_ and CFU ml^-1^ by serially diluting in saline containing 0.05% tween-80 and plating on 7H10 + OADC plates.

*DNA manipulation, plasmid construction, and gene deletions*. DNA was propagated, amplified, digested, ligated, and transformed using standard protocols ([4](#_ENREF_4)). Genes were amplified using the primers and template genomic DNA listed in Table S1. Amplicons were digested with the enzymes indicated in the descriptions of the oligonucleotides. The nucleotide sequence of all constructs was verified prior to their use. Plasmids based on pTipQc2 and pMV361.apr were electroporated into RHA1 and *M. smegmatis*, respectively, as previously described ([1](#_ENREF_1), [5](#_ENREF_5)).

Mycobacterial genes were deleted using homologous recombination ([6](#_ENREF_6)). Allelic exchange substrate (AES) constructs were generated using the oligonucleotides listed in Table S1 to amplify the up- and downstream regions of the genes to be deleted and cloning them on either side of the *hyg^R^* cassette in pYUB854. The linearized AES was electroporated into *Mtb* and *M. smegmatis* harboring pJV53. In RHA1, mutants were obtained using a SacB-based selection as previously described ([7](#_ENREF_7)). Briefly, pK18-derived plasmids were electroporated into *E. coli* S17.1 and then conjugated into RHA1. After the second recombination, kanamycin-sensitive/sucrose-resistant colonies were screened and confirmed using PCR (Fig. S5).

*Macrophage infections*. THP-1 cells (American Type Culture Collection, TIB-202), were cultured in GIBCO® RPMI 1640 (Thermo Fisher Scientific) supplemented with 10% fetal bovine serum, 2 mM L-glutamine, and 1 mM sodium pyruvate and were maintained between 2 and 5 x 10^5^ cells ml^-1^. *Mtb* strains were grown to late log phase in Middlebrook 7H9 supplemented with OADC, aliquots were frozen at -80°C, and CFU ml^-1^ was enumerated. THP-1 cells were seeded in 24-well flat-bottom tissue culture plates and allowed to adhere in the presence of 50 ng ml^-1^ PMA for 48 hours at 37°C in a humidified, 5% CO_2_ atmosphere. Cells were washed to remove PMA and incubated for a further 48 hours prior to infection. Bacteria were added to THP-1 cells at an MOI of 1:1 for 6 hours. Cells were washed three times to remove extracellular bacteria and were then incubated for 7 days. At each time point, THP-1 cells were lysed by adding 0.06% SDS. Bacteria were then serially diluted in saline containing 0.05% tween-80, and plated on Middlebrook 7H10 with OADC for enumeration.

*Protein Production and Purification.* Proteins were produced in and purified from *E. coli* Rosetta 2 pLysS (IpdF_Mtb_), *E.coli* BL21 (DE3) (MBP-IpdC_DOC21_) or RHA1 (EchA20_RHA1_, FadA6_Mtb_ and IpdAB_RHA1_). Cells were grown on LB supplemented with carbenicillin, ampicillin or chloramphenicol at 50, 100 and 34 μg ml^-1^, respectively, as appropriate. Single colonies of freshly transformed cells were used to inoculate 50 ml growth medium and incubated overnight at 37^o^C, 200 rpm (*E. coli*) or 30^o^C, 200 rpm (RHA1). Ten ml of overnight culture was used to inoculate 1 l fresh medium. At an OD_600_ of ~0.6, inducer was added (0.5 mM IPTG for *E. coli*; 10 μg ml^-1^ thiostrepton for RHA1) and cultures were incubated for an additional 16 h, then cells were harvested by centrifugation. For *E. coli* cultures, this 16 h incubation was done at 25^o^C. In addition, *E. coli* cultures producing MBP-IpdC_DOC21_ were supplemented with 0.1% glucose to repress intracellular expression of amylases. Cell pellets were stored at -80^o^C until use.

To purify the various proteins, cell lysis buffers contained 2 U ml^-1^ DNaseI and one tablet protease inhibitor cocktail (Roche). *E. coli* cells were lysed using five passages through an Avestin Emulsiflex-05 homogenizer operated at 10,000 p.s.i. and RHA1 cells were lysed using an MP Biomedicals FastPrep-24 bead beater (five rounds of 40 s). Cell lysates were clarified by ultracentrifugation (40000 × g, 45 min at 4^o^C) then filtered through a 0.45 μm membrane. Proteins were buffer-exchanged and concentrated using a Centricon 30 K (Millipore) or an Amicon Stirred Cell (Millipore) equipped with a 30 K regenerated cellulose Ultrafiltration Membrane (Millipore), then flash frozen in liquid nitrogen. Protein preparations were evaluated using SDS PAGE.

To purify IpdF_Mtb_, pellets of *E.coli* Rosetta 2:pETRv3559c were suspended in 25 ml 50 mM sodium phosphate, pH 8.0, 10% glycerol. The clarified lysate was loaded onto 3 ml Ni-Sepharose 6 Fast Flow resin (GE Healthcare) and IpdF_Mtb_ was eluted using a gradient of 10-500 mM imidazole in 50 mM sodium phosphate, pH 8.0 according to the manufacturer’s protocol. Fractions containing IpdF_Mtb_ were pooled, dialyzed overnight against 25 mM HEPES, pH 7.5, 50 mM KCl and 10% glycerol, and concentrated to ~15 mg ml^-1^.

To purify MBP-IpdC_DOC21_, pellets of *E.coli* Rosetta2*:* pMALDOC21 were suspended in 30 ml 50 mM Tris, pH 8.0, 100 mM NaCl. The clarified lysate was loaded onto a 15 ml column of amylose resin (New England Biolabs). The resin was washed with ~75 ml 50 mM Tris, pH 8.0, 100 mM NaCl (Buffer A), then MBP-IpdC_DOC21_ was eluted using ~75 ml Buffer A containing 20 mM maltose. Fractions containing >70% MBP-IpdC_DOC21_ were pooled, concentrated to ~3 ml and exchanged into Buffer A. MBP-IpdC_DOC21_ was precipitated using a final concentration of 1.75 M ammonium sulfate in Buffer A. The white precipitate was collected via centrifugation (4000 × g, 5 min, 4^o^C), washed with fresh Buffer A containing 1.75 M ammonium sulfate and solubilized in 1 ml Buffer A.

To purify EchA20_RHA1_, FadA6_Mtb_, and IpdAB_RHA1_, pellets RHA1: pTipR1EchA20, RHA1:pTipFadA6, RHA1:pTipR1IpdAB, respectively, were suspended in 25 ml 50 mM sodium phosphate, pH 8.0 containing 300 mM NaCl and 10 mM imidazole. Clarified lysates were loaded onto 2 ml Ni-Sepharose G Fast Flow resin (GE Healthcare) and eluted using a gradient of 10-500 mM imidazole according to the manufacturer`s protocol. Fractions containing the desired protein were pooled and dialyzed overnight against either 25 mM HEPES, pH 7.5 and 50 mM NaCl (FadA6_Mtb_ and IpdAB_RHA1_) or 25 mM HEPES, pH 7.5, 300 mM NaCl, 1 mM MgCl_2_, 1 mM NaHCO_3­_ and 10% glycerol (EchA20_RHA1_). Proteins were concentrated to ~10 mg ml^-1^.

*Preparation of Steroid Metabolites and CoA Thioesters.* HIP and HIP-CoA were prepared as described previously ([1](#_ENREF_1), [8](#_ENREF_8)). *p*-Coumaroyl-CoA was synthesized as described using CouL ([9](#_ENREF_9)) and was quantified spectrophotometrically in 50 mM sodium phosphate, pH 7.1 (ε_333_ = 21,000 M^-1^ cm^-1^) ([10](#_ENREF_10)). Propionyl-CoA and succinyl-CoA were synthesized from their corresponding anhydride as previously described ([11](#_ENREF_11)). 5α-OH HIC-CoA was synthesized from 5α-OH HIC using the mixed anhydride method ([12](#_ENREF_12)). Total synthesis of the 5-OH HICs will be published elsewhere. Briefly, 5 mg 5αOH-HIC dissolved in 0.5 ml dry THF was reacted with 10 μl ethylchloroformate and 26 μl triethylamine (TEA) for 20 min at room temperature. The resulting mixed anhydride was filtered through glass wool into 15 mg of CoASH dissolved in 1 ml THF:water (2:3, v:v), adjusted with NaOH to ~pH 8, and reacted at 37^o^C for 1 h. The reaction was stopped with the addition of 20 μl acetic acid and THF was removed under nitrogen. Typical mole yields were 80%.

CoA thioesters were HPLC-purified using a Luna 3 µm PFP(2) 50 × 4.6 mm column (Phenomenex) equilibrated with 0.1 M ammonium acetate, pH 4.5. CoA thioesters were eluted using a 20 min linear gradient of 0 to 90% methanol in 0.1 M ammonium acetate, pH 4.5. The eluate was monitored at 260 nm. Methanol was removed under N_2_ and compounds stored at -80°C. CoASH, acetyl-CoA, propionyl-CoA succinyl-CoA, 5α-OH HIC-CoA, and HIP-CoA were quantified at 260 nm using an ε_260_ of 11,900 M^-1^ cm^-1^ in 50 mM sodium phosphate, pH 7.1. The identities of the CoA thioesters were verified by LC/MS/MS using a Zorbax SB300-C18 150 × 0.075 mm column (Agilent Technologies) and an Agilent 6550 ToF mass spectrometer operated as described in Materials and Methods.

*Preparation of COCHEA-CoA.* COCHEA-CoA was obtained from Δ*ipdAB* RHA1 using a protocol similar to that described above in the *preparation of steroid metabolites* to prepare CoA thioester metabolites for MS analysis with the following modifications. Phospholipid-free CoA metabolomes prepared from 10 × 4 l of *ΔipdAB* RHA1 were pooled, dried using a SpeedVac^TM^, suspended in 1 ml water and filtered using 0.2 μm PTFE membrane. COCHEA-CoA was purified from the CoA metabolome using a HP1100 series HPLC (Agilent Technologies) equipped with a Luna 3u PFP(2) 50 x 4.6 mm column (Phenomenex) operated at 1 ml min^-1^ and separated over a gradient of 0-60% methanol (90%) in 100 mM ammonium acetate, pH 4.5 over 12 min. COCHEA-CoA eluted at 8.4 min as a single species with a λ_max_ = 250 nm. HPLC-purified COCHEA-CoA fractions were dried using a SpeedVac^TM^, suspended in 2 ml water and dialyzed against 2 l of water using a 100-500 Da cellulose ester dialysis membrane (Spectrum Laboratories Inc.). Desalted COCHEA-CoA was dried using a SpeedVac^TM^, washed and dried twice in deuterated methanol, then dissolved in 450 μl deuterated water (D_2_O). The final concentration of COCHEA-CoA was estimated to be 600 μM as determined using an HPLC standard curve of CoASH (ε_260_ = 11.9 mM^-1^ cm^-1^) and using an extinction coefficient of 16.4 mM^-1^ cm^-1^ due to the additional absorbance from a double bond ([13](#_ENREF_13)). Each biotransformation, using cells from 4 l of culture, yielded ~48 nmol of COCHEA-CoA. COCHEA-CoA was confirmed via LC/MS/MS prior to NMR.

*Preparation of MOODA*. MOODA was purified from the supernatant of cholesterol-incubated Δ*fadE32* *M. smegmatis* as follows. Four × 1 l cultures were grown to mid log (OD_600_ = 0.6) in 7H9 media + 0.5% Tween 20 + 0.2% glycerol and harvested by centrifugation (4000 × g, 20 min at 16^o^C). Cells were washed once using M9 salts, suspended in 200 ml M9 salts, 2 mM MgSO_4_, 0.1 mM CaCl_2_ and 0.5 mM cholesterol, split into 2 × 100 ml, then incubated at 37^o^C for 24 h in 250 ml baffled flasks. Cells were harvested by centrifugation and discarded. The supernatant was collected, acidified to ~pH 2 using HCl and extracted 3× with 1:1(v:v) ethyl acetate. The organic phases were pooled, dried over anhydrous MgSO_4_ and filtered through Whatman paper. Ethyl acetate was removed using a rotavap. The oily residue was dissolved in water and brought to pH 7 with NaOH. MOODA was purified using a Strata-X-A strong anionic exchange solid phase extraction column (Phenomenex) according to the manufacturer’s protocol. GC/MS analysis indicated that MOODA was >95% homogeneous. The yield of MOODA was ~125 μg l^-1^. For NMR characterization, ~0.5 mg MOODA was dried using a SpeedVac^TM^, washed twice with deuterated methanol (MeOD), and dissolved in 500 μl D_2_O.

*NMR characterization of metabolites.* ^1^H-NMR, ^1^H-^13^C HMBC, ^1^H-^13^C HSQC, ^1^H-^1^H COSY, and ^1^H-^1^H TOCSY spectra were recorded at 25^o^C using a Bruker 850 MHz NMR spectrometer. ^1^H-NMR spectra were recorded before and after each experiment to ensure no degradation had occurred during data collection. NMR data were analyzed using the ACD/NMR Processor v12.0 Academic Edition software.

*Enzyme activities*. Assays using purified enzymes were performed in a final volume of 100 μl containing 10 mM sodium phosphate, pH 8.0, 100 μM 5α-OH HIC-CoA, 100 μM NAD^+^, 5 μM FMN and 2 μM of each relevant enzyme. Assays containing FadA6_Mtb_ also contained 50 μM CoASH. Reactions were incubated at 37^o^C for 1 h. Proteins were removed before HPLC and MS analysis by the addition of 200 μl acetonitrile + 5% acetic acid. Volatile solvents were removed using a SpeedVac^TM^ concentrator and precipitated proteins were pelleted by centrifugation (16,000 × g, 5 min, 4^o^C). Samples were filtered through a 0.2 μm membrane, diluted 2:1 in water and run on an HP1100 series HPLC equipped with a Luna 3u PFP(2) column. The eluate was monitored at 260 nm. The identity of new peaks was confirmed by LC/MS/MS as described above.

*Bioinformatic analyses*. The amino acid sequences of Rings C/D catabolic enzymes were obtained from the NCBI using their *Mtb* H37Rv gene loci (Table 1). These sequences were used to search for homologs in the genomes of RHA1, *M. smegmatis* MC^2^155, *C. testosteroni* CNB-2, and *S. denitrificans* DSM 18526 using BLAST-P. Best hits were used to search the *Mtb* H37Rv genome to evaluate whether they were reciprocal best hits. Closest characterized homologs were determined by using the amino acid sequences of *Mtb* Rings C/D catabolic enzymes to search against the Protein Data Bank database using BLAST-P and manually identifying the best characterized result for each enzyme. The EchA phylogenetic tree (Fig. S4) was generated using MUSCLE and the sequences of enzymes shown in the tree ([14](#_ENREF_14)).

**RESULTS**

*Purification of KstR2 regulon enzymes.* IpdC_DOC21_, IpdF_Mtb_, EchA20_RHA1_, IpdAB_RHA1_, and FadA6_Mtb_ were purified to apparent homogeneity (Fig. S5). Typical yeilds from 1 l of bacterial cell cultures were 16, 90, 20, 40, and 25 mg, respectively.

*Analytical data of metabolites.*

**CoASH**


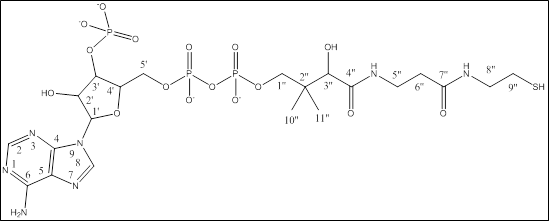


ɛ_260nm, pH 7.0_= 11.9 mM^-1^ cm^-1^ ([1](#_ENREF_1)).

**[M+H]^+^** (ESI-MS/MS) 768.1210 (261.1269, 428.0364); C_21_H_37_N_7_O_16_P_3_S^+^ ; λ_max_= 258 nm

**^1^H-NMR (850 MHz, D_2_O):** δ= 8.69 (s,H,8), 8.29 (s,H,4), 6.19 (d,H,1’),4.85 (s,H,3’’), 4.59 (s,H,4’), 4.20 (s,2H,5’), 4.03 (s,2H,2’), 3.81-3.35 (m,8H), 2.59 (t,2H,6’’), 2.51 (t,2H,5’’), 0.90 (s,3H,10’’), 0.78 (s,3H,11’’)

**^13^C-NMR (from ^1^H-^13^C HMBC/HSQC; 850 MHz, D_2_O)**: δ= 178 (C4’’), 177 (C7’’), 158 (C6), 155 (C2), 153 (C4), 144 (C8), 122 (C5), 89 (C1’), 86 (C4’), 77, 75, 73, 68 (C5’^c^), 45 (C8’’), 41 (C2’’), 38 (C5’’^c^), 38 (C6’’^c^), 31 (C9’’^c^), 23 (C11’’), 21 (C10’’)


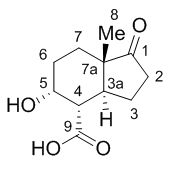
**3a*α*-*H*-4*α*(carboxyl)-5α-hydroxy-7a*β*-methylhexahydro-1-indanone (5α-OH HIC)**

MP: 154.4-156^o^C (colorless liquid); λ_max_= 258 nm

**[M+H]^+^** (ESI-MS/MS) 962.2159 (455.2209, 428.0368); C_32_H_51_N_7_O_19_P_3_S^+^

**^1^H-NMR (400 MHz, MeOD)**: δ= 4.30-4.27 (m,H,5), 2.67 (dd, *J* =12.2,2.8 Hz,H,4), 2.48-2.41 (m,H,2), 2.31-2.20 (m,2H,3a,3), 2.16-2.09 (m,H,2), 1.84-1.80 (m,2H,6), 1.68-1.58 (m,2H,3,7), 1.53-1.49 (m,H,7), 0.91 (s,3H,8)

**^13^C-NMR (100 MHz, MeOD)**: δ= 222 (C1), 177 (C9), 68 (C5), 49 (C7a), 48 (C4) 41 (C3a), 36 (C2), 30 (C6), 27 (C7), 24 (C3), 13 (C8)

**2‑TMS-5α-OH HIC** GCMS *R_t_* = 10.72 min. MS (70 eV, EI); *m/z*: 356 (4%), 341 (54%), 300 (11%), 147 (100%), 73 (78%)

**3a*α*-*H*-4*α*(carboxyl)-5β-hydroxy-7a*β*-methylhexahydro-1-indanone (5β-OH HIC)**

**
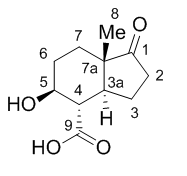
**

MP: 193-195^o^C (colorless liquid); λ_max_= 258 nm

**[M+H]^+^** (ESI-MS/MS) 962.2170 (455.2209, 428.0367); C_32_H_51_N_7_O_19_P_3_S^+^

**^1^H-NMR (400 MHz, MeOD)**: δ= 3.66 (ddd, *J* = 11.2, 10.0,5.5 Hz,H,5), 2.52-2.45 (m,2H,4,2), 2.21-2.11 (m,H,2), 1.96-1.89 (m,H,6), 1.86-1.67 (m,4H,3a,3,7), 1.65-1.57 (m,H,6), 1.41-1.35 (m,H,7), 0.97 (s,3H,8)

**^13^C-NMR (100 MHz, MeOD)**: δ= 221 (C1), 177 (C9), 73 (C5), 52 (C4), 48 (C7a), 47 (C3a), 37 (C2), 31 (C6), 30 (C7), 23 (C3), 14 (C8)

**2‑TMS-5β-OH HIC** GCMS *R_t_* = 10.70 min. MS (70 eV, EI); *m/z*: 356 (9%), 341 (47%), 300 (15%), 147 (100%), 73 (96%)

**
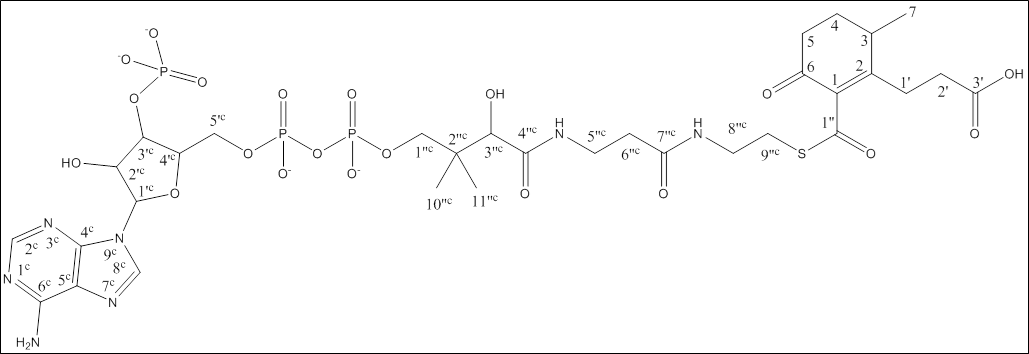
2-(2-carboxyethyl)-3-methyl-6-oxocyclohex-1-ene-1-carboxyl-CoA (COCHEA-CoA)**

ɛ_260nm, pH 7.0_= 16.4 mM^-1^ cm^-1^ (calculated).

**[M+H]^+^** (ESI-MS/MS) 976.1960 (469.1994, 428.0358); C_32_H_49_N_7_O_20_P_3_S^+^; λ_max_= 250 nm

**^1^H-NMR (850 MHz, D_2_O):** δ= 8.64 (s,H,8^c^), 8.23 (s,H,4^c^), 6.19 (d,H,1’^c^),4.79 (s,H,3’’^c^), 4.63 (s,H,4’^c^), 4.23 (s,2H,5’^c^), 4.00 (s,2H,2’^c^), 3.58-3.39 (m,7H), 3.16 (m,2H, 9’’^c^), 2.73 (m,H, 3), 2.65 (m,2H, 5,1’), 2.59 (t,2H, 2’), 2.44 (t,H,5’’^c^), 2.38 (m,H,5), 2.10 (m,H,4), 1.81 (m,H,4) 1.23 (d *^3^J=*9 Hz,3H,7), 0.90 (s,3H,10’’^c^), 0.78 (s,3H,11’’^c^); ^c^ denotes CoA moiety

**^13^C-NMR (from ^1^H-^13^C HMBC/HSQC; 850 MHz, D_2_O)**: δ= 203 (C6), 201 (C1’’), 182 (C3’), 177 (C4’’^c^), 177 (C7’’^c^), 175 (C2), 156 (C6^c^), 155 (C2^c^), 152 (C4^c^), 142 (C8^c^), 139 (C1), 121 (C5^c^), 89 (C1’^c^), 83 (C4’^c^), 77 (C3’’^c^), 75 (C2’^c^), 73 (C3’^c^), 68, 62 (C5’^c^), 41 (C2’’^c^), 38 (C5’’^c^), 38 (C6’’^c^), 36 (C5), 36 (C1’), 35 (C3), 32 (C2’), 31 (C9’’^c^), 31 (C4), 24 (C11’’^c^), 21 (C10’’^c^), 19 (C7);  ^c^ denotes CoA moiety

^
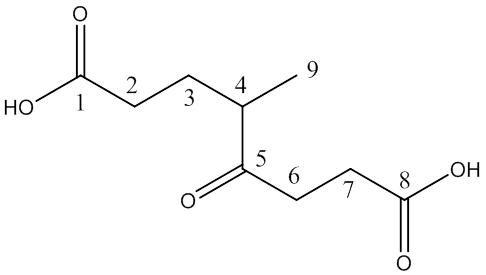
^**4-Methyl-5-oxo-octanedioc acid (MOODA)**

**^1^H NMR (850 MHz, D_2_O)**: δ= 2.97 (m,1H,6^x^), 2.89 (m,1H,6^y^), 2.77 (m,1H,4), 2.61 (t,2H,8), 2.37 (t,2H,2), 1.94 (m,1H,3^x^), 1.69 (m,1H,3^y^), 1.13 (d,*^3^J=* 7.1 Hz,3H, 9)

**^13^C NMR (from ^1^H-^13^C HMBC/ HSQC; 850 MHz, D_2_O)**: δ= 221 (C5), 180 (C1), 179 (C8), 48 (C4), 38 (C6), 32 (C2), 30 (C7), 29 (C3), 18 (C9)

2‑TMS-MOODA GCMS *R_t_* = 10.02 min. MS (70 eV, EI); *m/z*: 346 (1%), 331 (12%), 241 (14%), 173 (100%), 125 (32%), 73 (82%)

**4-Methyl-5-oxo-octanedioyl-CoA (MOODA-CoA)**

**[M+H**] 952.1960 (445.1999, 428.0371) m/z; C_30_H_49_N_7_O_20_P_3_S^+^_;_ λ_max_ = 258 nm

^
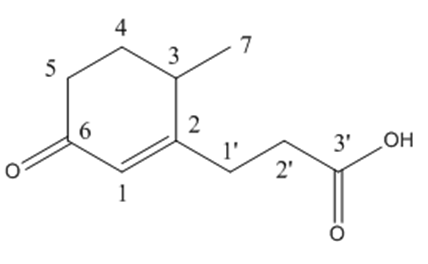
^**2-(2-carboxyethyl)-3-methyl-6-oxocyclohex-1-ene-1**

**1‑TMS-COCHE** GCMS *R_t_* = 9.32 min. MS (70 eV, EI); *m/z*: 254 (83%), 239 (25%), 221 (59%), 197 (40%), 137 (92%), 122 (100%)

**^1^H-NMR (600 MHz, D_2_O):** δ= 5.8 (s,1H,1), 2.6 (m,6H,5,3’,1’), 2.4 (m,1H,3), 2.1 (m,1H,4^x^), 1.8 (m,1H,4^y^), 1.2 (d,*^3^J=* 8 Hz,3H,7)

**^13^C-NMR (from HSQC/HMBC; 600 MHz, D_2_O):** δ= 205 (C6), 180 (C3’), 174 (C2), 128 (C1), 36 (C5), 34 (C3), 34 (C2’), 32 (C4), 32 (C1’), 17 (C7)

**(7aS)-7a-Methyl-1,5-dioxo-2,3,5,6,7,7a-hexahydro-1H-indene-4-carboxyl-CoA (HIEC-CoA)**


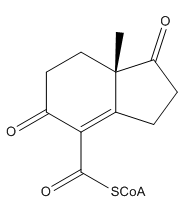


**[M+H]** 958.1899 (451.1914, 428.0378) m/z; C_32_H_47_N_7_O_19_P_3_S^+^_;_ λ_max_ = 253 nm

**REFERENCES**

1. Casabon I, Crowe AM, Liu J, Eltis LD. 2013. FadD3 is an acyl-CoA synthetase that initiates catabolism of cholesterol rings C and D in actinobacteria. Mol Microbiol 87:269-283.

2. Upton AM, McKinney JD. 2007. Role of the methylcitrate cycle in propionate metabolism and detoxification in *Mycobacterium smegmatis*. Microbiology 153:3973-3982.

3. Griffin JE, Gawronski JD, DeJesus MA, Ioerger TR, Akerley BJ, Sassetti CM. 2011. High-resolution phenotypic profiling defines genes essential for mycobacterial growth and cholesterol catabolism. PLoS Pathog 7 e1002251.

4. Sambrook J, Russell DW. 2001. Molecular Cloning: A Laboratory Manual. Cold Spring Harbor Laboratory Press.

5. Ramon-Garcia S, Ng C, Jensen PR, Dosanjh M, Burian J, Morris RP, Folcher M, Eltis LD, Grzesiek S, Nguyen L, Thompson CJ. 2013. WhiB7, an Fe-S-dependent transcription factor that activates species-specific repertoires of drug resistance determinants in actinobacteria. J Biol Chem 288:34514-28.

6. van Kessel JC, Marinelli LJ, Hatfull GF. 2008. Recombineering mycobacteria and their phages. Nature reviews Microbiology 6:851-857.

7. van der Geize R, Hessels GI, van Gerwen R, van der Meijden P, Dijkhuizen L. 2001. Unmarked gene deletion mutagenesis of *kstD*, encoding 3-ketosteroid ∆1-dehydrogenase, in *Rhodococcus erythropolis* SQ1 using *sacB* as counter-selectable marker. FEMS Microbiol Lett 205:197-202.

8. Casabon I, Zhu SH, Otani H, Liu J, Mohn WW, Eltis LD. 2013. Regulation of the KstR2 regulon of *Mycobacterium tuberculosis* by a cholesterol catabolite. Mol Microbiol 89:1201-1212.

9. Otani H, Lee Y-E, Casabon I, Eltis LD. 2014. Characterization of *p*-hydroxycinnamate catabolism in a soil Actinobacterium. J Bacteriol 196:4293-4303.

10. Rautengarten C, Baidoo E, Keasling JD, Scheller HV. 2010. A simple method for enzymatic synthesis of unlabeled and radiolabeled hydroxycinnamate-CoA. Bioenerg Res 3:115-122.

11. Peter DM, Vogeli B, Cortina NS, Erb TJ. 2016. A Chemo-Enzymatic Road Map to the Synthesis of CoA Esters. Molecules 21.

12. Thomas ST, VanderVen BC, Sherman DR, Russell DG, Sampson NS. 2011. Pathway profiling in *Mycobacterium tuberculosis*: elucidation of cholesterol-derived catabolite and enzymes that catalyze its metabolism. J Biol Chem 286:43668-78.

13. Ho NA, Dawes SS, Crowe AM, Casabon I, Gao C, Kendall SL, Baker EN, Eltis LD, Lott JS. 2016. The Structure of the Transcriptional Repressor KstR in Complex with CoA Thioester Cholesterol Metabolites Sheds Light on the Regulation of Cholesterol Catabolism in *Mycobacterium tuberculosis*. J Biol Chem 291:7256-66.

14. Edgar RC. 2004. MUSCLE: multiple sequence alignment with high accuracy and high throughput. Nucleic Acids Research 32:1792-1797.

**SUPPLEMENTAL TABLES**

Table S1. Strains, plasmids, and oligonucleotides used in this study.

Table S2. List and characterization of CoA thioesters in *Mtb*, RHA1, *M. smegmatis* and Δ*ipdAB* mutants.

Table S3. List of targeted MRMs followed for each CoA metabolome analyzed by LC/MS.

**SUPPLEMENTAL FIGURE LEGENDS**

**Figure S1. Growth and CoA metabolites of RHA1 strains.** Growth of WT::pTip-Qc2 (blue), Δ*ipdAB* ::pTip-Qc2 (red), Δ*ipdAB*:: pTipCoL51 (red, dashed), Δ*ipdC*::pTipQc2 (green) and Δ*ipdC::* pTipRv3553 (green, dashed) on: (A) 10 mM pyruvate; (B) 1 mM cholesterol; (C) 1.5 mM HIP; and (D) 1 mM HIP plus 10 mM pyruvate. (E) Depletion of HIP by RHA1 strains, color-coded as in growth curves as measured by GC/MS and reported as % of initial levels. Data are the mean of triplicates. Error bars show standard deviation (F) LC/MS chromatograms of CoA metabolites extracted from WT (blue) and Δ*ipdAB* (red) RHA1 incubated with cholesterol. Numbers correspond to (1) CoASH; (2) acetyl-CoA; (3) propionyl-CoA; and (4) COCHEA-CoA. IS = internal standard. Data for panel D were acquired using a BioScreen C (Growth Curves USA).

**Figure S2. Growth and CoA metabolites of *ΔipdC Mtb.*** WT (black), *ΔipdC* (red), *ΔipdC::ipdC* (blue) *Mtb* Erdman were grown on (A) 1 mM cholesterol; (B) 0.2% glycerol; or (C) 0.5 mM cholesterol and 0.2% glycerol. (D) CoA metabolome of *ΔipdC* (red) and WT *Mtb* (blue) incubated with 0.5 mM cholesterol. Arrows indicate the peaks corresponding to the 5-OH HIC-CoA in the *ΔipdC* RHA1 CoA metabolome.

**Figure S3. CoA thioesters and metabolites produced by *M. smegmatis* strains**. (A) WT and (B) Δ*ipdAB* cells were incubated with each of cholesterol (blue) and glycerol (grey). Numbers represent (1) CoASH; (2) acetyl-CoA; (3) succinyl-CoA; (4) propionyl-CoA; (5) unidentified CoA thioester 838 m/z; (6) unidentified CoA thioester 852 m/z; and (7) COCHEA-CoA. (C) GC/MS of culture supernatants of cholesterol-incubated *M. smegmatis* strains. Cells of the indicated strains were incubated with 0.5 mM cholesterol. Insets show structures of TMS-derivatized 5α-OH HIC and 2-(2-carboxyethyl)-3-methyl-6-oxocyclohex-1-ene-1. * indicates unidentified compounds present in all *M. smegmatis* extracts that is unrealted to cholesterol catabolism.

**Figure S4. Phylogenetic tree of enoyl-CoA hydratases (EchAs) and MenB in *Mtb.*** Cluster containing EchA20 and MenB_Mtb_ is highlighted with a red oval.

**Figure S5. Electrophoretic analyses of gene deletion mutants and purified proteins.** (A) PCR confirmation of Δ*ipdAB* in RHA1 and Mtb; Δ*ipdC* in RHA1 and Mtb; Δ*ipdF*, Δ*echA20*, and Δ*fadE32* in *M. smegmatis* using the listed primer sets (Table S1). (B) SDS PAGE loaded, from left to right, with 0.5 μg each of, MBP-IpdC_DOC21_, IpdF_Mtb_, EchA20_RHA1_, IpdAB_RHA1_, and FadA6_Mtb_. Purified proteins are flanked by molecular weight standards.

**Figure S6. LC/MS of CoA thioester standards**. (A) Peaks correspond to 25 pmol CoASH (light blue), acetyl-CoA (purple), propionyl-CoA (dark blue), 5α-OH HIC-CoA (yellow), HIP-CoA (green), and *p*-coumaroyl-CoA (red). (B) Representative standard curves for authentic CoA thioesters. Data points correspond to [M+1]=>-507 (blue) and [M+1]=> 428 (red) transitions. Lines represent best fit linear regression as follows: CoASH (768=>261, y = 667.34x - 1699.3, R^2^ = 0.9633; 768=>428, y = 350.09x + 157.39, R^2^ = 0.9892); acetyl-CoA (810=>303, y = 1167.7x - 2786.8, R^2^ = 0.9803; 810=>428, y = 254.43x - 365.49, R^2^ = 0.9742); HIP-CoA (988=>481, y = 447.29x - 1099, R^2^ = 0.9734; 988=>428, y = 97.542x - 192.62, R^2^ = 0.9735); *p-*coumaroyl-CoA (914=>407, y = 428.8x + 19.13,R^2^ = 0.9786). (C) Collision Energy Dissociation (CID) optimization. Peak intensities of 50 pmol authentic CoA thioester standards for [M+H]=> -507 (blue) and [M+H]=> 428 (red) transitions over different CID voltages.
