# Supplementary material for: Catabolism of the Last Two Steroid Rings in Mycobacterium tuberculosis and Other Bacteria
Source: mBio. 2017 Apr 4;8(2):e00321-17. doi: 10.1128/mBio.00321-17 (PMC5380842; doi:10.1128/mBio.00321-17)
Supplement: TABLE S3 [file mbo002173251st3.docx]

**Table S3. List of targeted MRMs followed for analysis of CoA metabolomes.**

| Parent Mass (m/z) | Fragment Mass (m/z) | Notes |
| --- | --- | --- |
| 1008 | 501 | [M+H] -507 m/z fragment |
| 1006 | 499 | [M+H] -507 m/z fragment |
| 1004 | 497 | [M+H] -507 m/z fragment |
| 996 | 489 | [M+H] -507 m/z fragment |
| 994 | 487 | [M+H] -507 m/z fragment |
| 992 | 485 | [M+H] -507 m/z fragment |
| 990 | 483 | [M+H] -507 m/z fragment |
| 988 | 481 | [M+H] -507 m/z fragment |
| 978 | 471 | [M+H] -507 m/z fragment |
| 976 | 469 | [M+H] -507 m/z fragment |
| 974 | 467 | [M+H] -507 m/z fragment |
| 968 | 461 | [M+H] -507 m/z fragment |
| 966 | 459 | [M+H] -507 m/z fragment |
| 964 | 457 | [M+H] -507 m/z fragment |
| 962 | 455 | [M+H] -507 m/z fragment |
| 960 | 453 | [M+H] -507 m/z fragment |
| 958 | 451 | [M+H] -507 m/z fragment |
| 956 | 449 | [M+H] -507 m/z fragment |
| 952 | 445 | [M+H] -507 m/z fragment |
| 950 | 443 | [M+H] -507 m/z fragment |
| 948 | 441 | [M+H] -507 m/z fragment |
| 946 | 439 | [M+H] -507 m/z fragment |
| 940 | 433 | [M+H] -507 m/z fragment |
| 938 | 431 | [M+H] -507 m/z fragment |
| 914 | 407 | [M+H] -507 m/z fragment |
| 868 | 361 | [M+H] -507 m/z fragment |
| 852 | 345 | [M+H] -507 m/z fragment |
| 838 | 331 | [M+H] -507 m/z fragment |
| 824 | 317 | [M+H] -507 m/z fragment |
| 810 | 303 | [M+H] -507 m/z fragment |
| 768 | 261 | [M+H] -507 m/z fragment |
| 1008 | 428 | [M+H] => 428 m/z fragment |
| 1006 | 428 | [M+H] => 428 m/z fragment |
| 1004 | 428 | [M+H] => 428 m/z fragment |
| 996 | 428 | [M+H] => 428 m/z fragment |
| 994 | 428 | [M+H] => 428 m/z fragment |
| 992 | 428 | [M+H] => 428 m/z fragment |
| 990 | 428 | [M+H] => 428 m/z fragment |
| 988 | 428 | [M+H] => 428 m/z fragment |
| 978 | 428 | [M+H] => 428 m/z fragment |
| 976 | 428 | [M+H] => 428 m/z fragment |
| 974 | 428 | [M+H] => 428 m/z fragment |
| 968 | 428 | [M+H] => 428 m/z fragment |
| 966 | 428 | [M+H] => 428 m/z fragment |
| 964 | 428 | [M+H] => 428 m/z fragment |
| 962 | 428 | [M+H] => 428 m/z fragment |
| 960 | 428 | [M+H] => 428 m/z fragment |
| 958 | 428 | [M+H] => 428 m/z fragment |
| 956 | 428 | [M+H] => 428 m/z fragment |
| 952 | 428 | [M+H] => 428 m/z fragment |
| 950 | 428 | [M+H] => 428 m/z fragment |
| 948 | 428 | [M+H] => 428 m/z fragment |
| 946 | 428 | [M+H] => 428 m/z fragment |
| 940 | 428 | [M+H] => 428 m/z fragment |
| 938 | 428 | [M+H] => 428 m/z fragment |
| 914 | 428 | [M+H] => 428 m/z fragment |
| 868 | 428 | [M+H] => 428 m/z fragment |
| 852 | 428 | [M+H] => 428 m/z fragment |
| 838 | 428 | [M+H] => 428 m/z fragment |
| 824 | 428 | [M+H] => 428 m/z fragment |
| 810 | 428 | [M+H] => 428 m/z fragment |
| 768 | 428 | [M+H] => 428 m/z fragment |
| 1252 | 428 | Negative Control^A^ |
| 1252 | 745 | Negative Control^A^ |
| 980 | 428 | Negative Control^A^ |
| 980 | 473 | Negative Control^A^ |
| 914 | 407 | Negative Control^A^ |
| 912 | 348 | Negative Control^A^ |
| 912 | 485 | Negative Control^A^ |
| 911 | 348 | Negative Control^A^ |
| 911 | 404 | Negative Control^A^ |
| 911 | 428 | Negative Control^A^ |
| 908 | 401 | Negative Control^A^ |
| 908 | 428 | Negative Control^A^ |
| 898 | 348 | Negative Control^A^ |
| 898 | 471 | Negative Control^A^ |
| 896 | 348 | Negative Control^A^ |
| 896 | 469 | Negative Control^A^ |
| 888 | 348 | Negative Control^A^ |
| 888 | 381 | Negative Control^A^ |
| 888 | 428 | Negative Control^A^ |
| 886 | 348 | Negative Control^A^ |
| 886 | 459 | Negative Control^A^ |
| 882 | 438 | Negative Control^A^ |
| 882 | 455 | Negative Control^A^ |
| 878 | 348 | Negative Control^A^ |
| 878 | 451 | Negative Control^A^ |
| 786 | 348 | Negative Control^A^ |
| 786 | 359 | Negative Control^A^ |
| 784 | 348 | Negative Control^A^ |
| 784 | 357 | Negative Control^A^ |
| 688 | 261 | Negative Control^A^ |
| 688 | 348 | Negative Control^A^ |
| 664 | 348 | Negative Control^A^ |
|  |  |  |
| ^A^ No transition predicted by proposed HIP degradation pathway. | | |
